# Facile Preparation of *N*-Glycosylated 10-Piperazinyl Artemisinin Derivatives and Evaluation of Their Antimalarial and Cytotoxic Activities

**DOI:** 10.3390/molecules23071713

**Published:** 2018-07-13

**Authors:** Yuet Wu, Silvia Parapini, Ian D. Williams, Paola Misiano, Ho Ning Wong, Donatella Taramelli, Nicoletta Basilico, Richard K. Haynes

**Affiliations:** 1 Department of Chemistry, The Hong Kong University of Science and Technology, Clear Water Bay, Kowloon, Hong Kong, China; dogbill002008@yahoo.com.hk (Y.W.); chwill@ust.hk (I.D.W.); 2 Department of Biomedical, Surgical and Dental Sciences (DiSBIOC), University of Milan, Via Pascal 36, 20133 Milan, Italy; silvia.parapini@unimi.it; 3 Department of Pharmacological & Biomolecular Sciences (DiSFeB), University of Milan, Via Pascal 36, 20133 Milan, Italy; Paola.misiano@unimi.it (P.M.); donatella.taramelli@unimi.it (D.T.); 4 Center of Excellence for Pharmaceutical Sciences, Faculty of Health Sciences, North-West University, Potchefstroom 2520, South Africa; 25904442@nwu.ac.za; 5 Inter University Center for Malaria Research, Italian Malaria Network, University of Perugia, 06100 Perugia, Italy

**Keywords:** artemisinins, artemisone, piperazine, *N*-glycosides, antimalarial activities, anti-tumour activities

## Abstract

According to the precepts that C-10 amino-artemisinins display optimum biological activities for the artemisinin drug class, and that attachment of a sugar enhances specificity of drug delivery, polarity and solubility so as to attenuate toxicity, we assessed the effects of attaching sugars to N-4 of the dihydroartemisinin (DHA)-piperazine derivative prepared in one step from DHA and piperazine. *N*-Glycosylated DHA-piperazine derivatives were obtained according to the Kotchetkov reaction by heating the DHA-piperazine with the sugar in a polar solvent. Structure of the D-glucose derivative is secured by X-ray crystallography. The D-galactose, L-rhamnose and D-xylose derivatives displayed IC_50_ values of 0.58–0.87 nM against different strains of *Plasmodium falciparum* (*Pf*) and selectivity indices (SI) >195, on average, with respect to the mouse fibroblast WEHI-164 cell line. These activities are higher than those of the amino-artemisinin, artemisone (IC_50_ 0.9–1.1 nM). Notably, the D-glucose, D-maltose and D-ribose derivatives were the most active against the myelogenous leukemia K562 cell line with IC_50_ values of 0.78–0.87 µM and SI > 380 with respect to the human dermal fibroblasts (HDF). In comparison, artemisone has an IC_50_ of 0.26 µM, and a SI of 88 with the same cell lines. Overall, the *N*-glycosylated DHA-piperazine derivatives display antimalarial activities that are greatly superior to *O*-glycosides previously obtained from DHA.

## 1. Introduction

**Figure 1:**
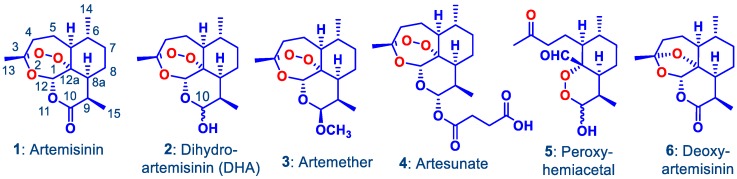
Artemisinin **1** and its clinically-used derivatives—the hemiacetal dihydroartemisinin (DHA) **2**, the lactol ether artemether **3**, and the hemiester artesunate **4**. The latter two are rapidly converted into DHA via metabolism or facile hydrolysis respectively. DHA rearranges irreversibly under physiological conditions into the peroxyhemiacetal **5** that in turn rearranges to the inert deoxyartemisinin **6**.

The derivatives **2**–**4** of artemisinin **1** (Figure 1) are the most widely used drugs for treatment of malaria caused mainly by the apicomplexan parasite *Plasmodium falciparum* (*Pf*). For treatment of non-severe malaria, combinations with longer half-life drugs known as artemisinin combination therapies (ACTs) are used [1]. Because of the peroxide group, artemisinins are thermally and chemically fragile, and show variable pharmacokinetic profiles and low bioavailability [2]. In particular, DHA **2** is labile due to an unzipping process driven by the unprotected hemiacetal hydroxyl group, it rearranges irreversibly under physiological conditions into the peroxyhemiacetal **5** that decomposes via a Kornblum-de la Mare process to the dead-end compound deoxyartemisinin **6** (Figure 1) [3]. The peroxyhemiacetal **5** is observed in plasma from patients treated with artesunate [4]. In this respect, it is noted that the antimalarial activity of DHA rapidly attenuates (half-life ~ 3 h) when incubated in human plasma or serum [5]. The instability also engenders problems during formulation and storage [6]. Again because of the hydroxyl group, DHA undergoes facile Phase I metabolism [7,8]. Artesunate **4** is rapidly hydrolyzed to DHA in vivo [9]. But because of the incipient protective effect of the ester group against first-pass metabolism, artesunate is a better source of DHA in plasma than DHA itself [10]. The facile metabolism of artemether **3** involving oxidative dealkylation of the methyl group, predominantly by CYP3A4, is reflected in the detection of DHA in subjects administered with artemether [11]. These artemisinins, especially DHA, display neurotoxicity in vitro and in vivo [12,13,14].

**Figure 2:**
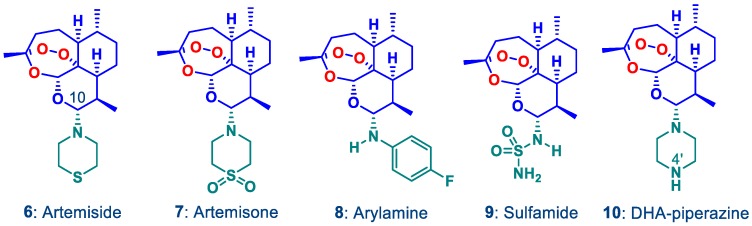
Amino-artemisinins bearing alkylamino, arylamino and sulfonylamino groups attached to C-10 of the artemisinin nucleus wherein the oxygen atom at C-10 in the current artemisinins (Figure 1) is replaced by the nitrogen atom. In terms of cost of goods, compounds **8**–**10** are the most economically obtained from DHA. The intent of the current work is to develop new compounds by attaching polar groups to N-4 in the piperazine derivative **10**.

However, resistance by malaria parasites to these derivatives, and to DHA in particular, now enormously complicates the task of global malaria control [15,16]. Thus, the development of new artemisinins that do not provide DHA and thereby supplant the current artemisinin derivatives is an urgent task. According to proposals based on the ADMET paradigm, [17] we prepared amino-artemisinin derivatives wherein the nitrogen atom of the amino group replaces the exocyclic oxygen atom attached to C-10 of the current clinical derivatives (Figure 2) [18,19,20,21]. All are significantly more active than the current clinical artemisinins against *Pf*. Whilst enhanced activity may be ascribed to improved pharmacokinetic parameters, this is better rationalized in terms of the conceptual model for mode of action that involves oxidation of reduced flavin cofactors important for modulating oxidative stress in the malaria parasite [22]. From a drug development perspective, the most advanced of the aminoartemisinins is artemisone **7** that is nine-fold more active than artesunate **4** against *Pf* in vitro and at least three-fold in in vivo [20,23,24]. Artemisone is not metabolized to DHA **2** but provides metabolites bearing unsaturation in the *S*,*S*-dioxothiomorpholino ring, and/or hydroxyl groups at C-5 or C-7 that also have potent antimalarial activity [20,25,26]. Unlike the current artemisinins, artemisone displays no clinically relevant autoinduction of metabolizing enzymes. [20,25,27,28] In comparison to DHA, [29] artemisone possesses a longer elimination half-life (~3 h vs. ~1 h), lower plasma clearance (~3.5 L/h/kg vs. ~5.4 L/h/kg) and a larger volume of distribution (14.5 L/kg vs. 7.7 L/kg), [27,28] and displays negligible neurotoxicity in vitro and in vivo [14]. Overall, artemisone is the only artemisinin derivative developed outside China that has progressed into a clinical trial against malaria. Artemisone has now been registered with the US FDA as an orphan drug planned for use in treatment of severe malaria via intravenous administration [30].

Artemisinins have also been tested for antitumour activities in vitro on cancer cell lines, in in vivo studies with animal models [31,32,33,34], and in pilot clinical trials [34,35]. However, as clinical treatment will require protracted treatment regimens, the DHA prodrug and the neurotoxicity problems essentially will, or should, proscribe use of the current clinical artemisinins. Artemisone **7** has antitumour properties, as well, and in combination with known anticancer agents, it possesses activities in vitro in the nanomolar to low micromolar range [36,37]. Combinations of artemisone with the anticancer drugs oxaliplatin, gemcitabine and thalidomide have a markedly potentiating effect on the action of each drug against human colorectal HCT116, colorectal adenocarcinoma SW480 and breast cancer MCF7 cells, an effect that is significantly greater than artemisinin itself. [36]. When artemisone is encapsulated into niosomes and solid lipid nanoparticles, the formulations display highly selective cytotoxicity towards melanoma A375 cells with negligible toxicity towards normal skin cells [38]. This thus allows for topical delivery of artemisone for treatment of melanoma [39].

Thus, 10-amino-artemisinins are desirable compounds, given their generally enhanced efficacies against malaria, superior metabolic profiles in the case of artemisone **7**, and promising activities against cancer. Of the various synthetic routes developed, a phase transfer approach involving DHA and a primary aromatic amine was the most expeditious in leading directly and with complete stereoselectivity to C-10 α-arylamino derivatives such as compound **8** [18]. For the others generally requiring more basic alkyl amine nucleophiles, phase transfer methods did not work, and *N*-glycosylation technology involving activation of the hydroxyl group in DHA by conversion into the ß-halide under anhydrous conditions had to be used. Treatment of the ß-halide in situ with the appropriate 2° amine nucleophile worked well in providing stereoselectively the α-alkylamino derivatives such as the α-thiomorpholino derivative artemiside **6** and its sulfone counterpart artemisone **7**, the sulfamide **9** and related compounds [19,20,21,40]. Of the various compounds earmarked for further development, the piperazine derivative **10** is attractive. Piperazine is a readily accessible, cheap synthon, and the free 2° amino group in the product allows for attachment of additional ligands to modulate drug properties. Thus, we have attached ferrocene containing groups to imprint redox-active behavior [41] and ligands based on cholesterol so as to enhance uptake through membranes such as the mycolic acid layer associated with *Mycobacterium tuberculosis* [42]. 

Here we use the piperazine derivative **10** as a template for preparing *N*-glycosylated artemisinin derivatives. Because of stereochemical issues involving the chiral, enantiomerically pure sugars, we had to secure configuration of the piperazine group at C-10 in **10**. Thereupon, the efficacies of the *N*-glycosylated derivatives would be evaluated in vitro against malaria and selected normal or immortalized cell lines.

**Figure 3:**
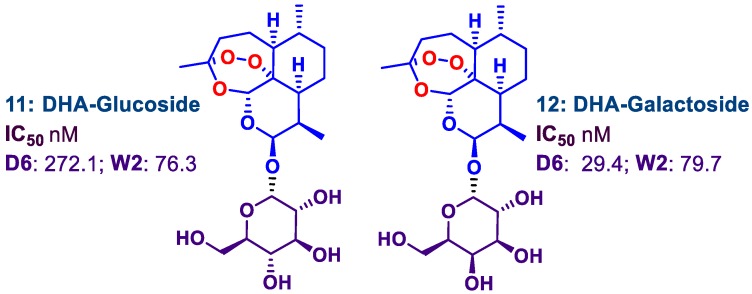
*O*-Glycosylated DHA derivatives and antimalarial activities against *Plasmodium falciparum* (*Pf*) D6 and W2 strains in vitro; cf. artemisinin **1**: IC_50_ D6 10.4 nM; W2 2.4 nM.

**Scheme 1:**
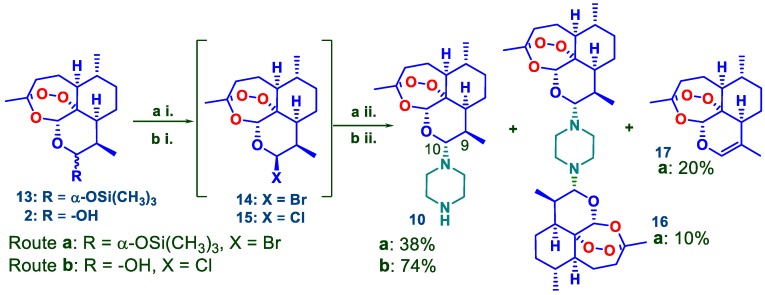
Preparation of DHA-piperazine derivative **10**. Route **a** i. DHA α-TMS ether **13** (1.40 mmol.), TMSBr (1.43 mmol., 1.02 equiv.), CH_2_Cl_2_, 0 °C, N2, 30 min; **a** ii. Solution from **a** i added to piperazine (5.61 mmol, 4.0 equiv.) in CH_2_Cl_2_, 0 °C—room temperature, 12 h, **10** 38%; **16** 10%; **17** 20%*.* Route **b** i. DHA **2** (3.52 mmol.), (COCl)_2_ (3.6 mmol., 1.02 equiv.), DMSO (0.35 mmol, 0.1 equiv.), toluene, N_2_, room temperature, 30 min; **b** ii. Solution from **b** i added to piperazine (14 mmol, 4 equiv.), CH_2_Cl_2_, 12 h; **10** 74%.

For malaria, it is not yet clear if the parasite possesses proteins with lectin-like activity (see below) capable of binding a sugar [43] and thus, it is uncertain how *N*-glycosylated artemisinins may express antimalarial activities. However, attachment of sugars to the hydroxyl group of DHA involving activation of DHA as the trimethylsilyl ether (cf. Scheme 1 below), treatment with the partially protected acetylated sugar under Lewis acid catalysis, and eventual hydrolysis of the acetylated sugar in the DHA precursor provided the DHA *O*-glycosides such as the *O*-glucosyl **11** and *O*-galactosyl **12** derivatives [44]. These were substantially less active than artemisinin **1** in vitro and essentially inactive in vivo (Figure 3). The more lipophilic peracetylated derivatives, that would be less capable of protein binding, however, were more active. The observation in general supports the theses that more polar artemisinin derivatives tend to be less active, but also less neurotoxic [20,45]. We are unaware of other studies involving assessment of antimalarial activities of glycosylated artemisinin derivatives.

In cancer pathogenesis, it is established that cell surface proteins called lectins, a family of carbohydrate-binding proteins, contribute to neoplastic transformation, tumor cell survival, angiogenesis and tumor metastasis [46]. Lectins have binding sites for galactose, *N*-acetyl glucosamine and others [47,48]. The feasibility of using carbohydrate ligands to target protein receptors at such binding sites, termed ‘glycotargeting’, is long established [49]. Because of the carbohydrate tag, the drug delivery system can be recognized, bound and internalized by endogenous lectin receptors at a tumour cell surface [50]. The technique has been extended to guide nanoparticles bearing fucose ligands and encapsulating an anticancer drug to lectin cell surface receptors on cancer cells [51]. Thus, through tagging artemisinins with ligands that overall may enhance specificity of drug delivery, we assess here the effects of attaching sugars known to bind to lectins to the piperazine unit of the DHA-piperazine derivative **10**.

## 2. Results and Discussion

### 2.1. Synthetic Chemistry: Preparation of DHA-Piperazine Derivative ***10***

The protocol for preparing artemiside **6** from the α-trimethylsilyl ether **13** of DHA and thiomorpholine [20,40] was applied to **13** by using piperazine, the caveat here being that with this difunctional nucleophile, formation of the bis-DHA piperazine derivative becomes a possibility. In the event, treatment of **13** firstly with trimethylsilyl bromide (TMSBr) in dichloromethane to generate the ß-bromide **14** in situ and then with anhydrous piperazine in the presence of triethylamine as base gave the polar DHA piperazine **10** (20%), the highly crystalline disubstituted piperazine **16** (10%) and the glycal **17** (20%). The last is a product common to these amination reactions; its formation is due to competing E1/E2 pathways involving the ß-bromide **14** as discussed elsewhere. [40] The bis-DHA piperazine **16** was prepared in higher yields (to 43%) by decreasing the amount of piperazine added to the solution of the ß-bromide **14**. Alternatively, yields of DHA piperazine **10** were increased (to 38%) by conducting inverse addition of the solution of the ß-bromide generated in situ to an excess of piperazine with omission of the triethylamine base (Route a, Scheme 1).

The foregoing method is unsatisfactory, and thus the newer process for optimization of the preparation of artemiside **6** and artemisone **7** was applied here. This involves use of the oxalyl chloride-catalytic dimethyl sulfoxide (DMSO) system to convert DHA into the ß-chloride **15** in toluene followed by treatment with the appropriate amine nucleophile. The products are obtained in good yields in scalable reactions that could be run in relatively concentrated solutions (to 1.2 M DHA) [40]. The role of the DMSO is crucial: in its absence in toluene, very little of the product is obtained. The mechanism likely involves activation of DMSO by oxalyl chloride according to the Swern oxidation to form a chlorosulfonium salt. This reacts with DHA to provide a sulfurane intermediate that collapses to the ß-chloride **15** with regeneration of DMSO [40]. Oxidation of the hydroxyl group in DHA as such does not interfere. In the event, inverse addition of the ß-chloride **15** solution in toluene to an excess of piperazine in dichloromethane now gave the DHA piperazine **10** in 74% yield. Alternatively, addition of a solution of piperazine (1 equiv.) in dichloromethane to the toluene solution of the ß-chloride **15** gave the bis-DHA piperazine **16** (35%) as the predominant product. 

**Figure 4:**
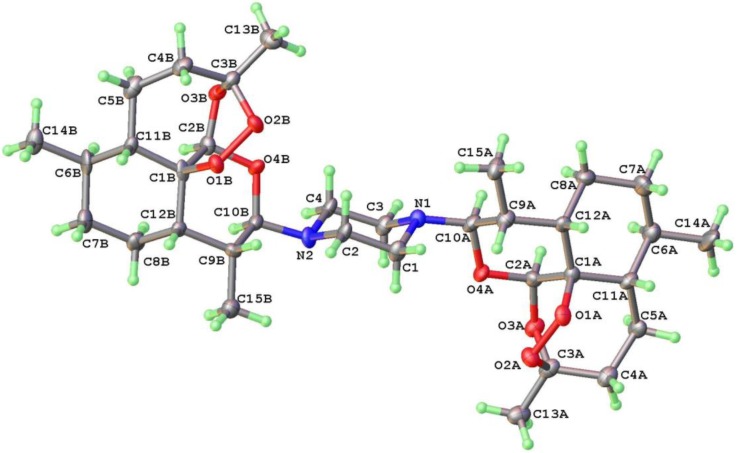
ORTEP plot from X-ray crystallographic structural determination of the 1,4-bis-DHA piperazine **16** indicating diaxial arrangement of H-9 and H-10 attached to each of the pyran rings in the respective artemisinin units, the chair piperazine and α-equatorial C-10 to N bonds. Numbering in the artemisinin A and B units is as for artemisinin in Figure 1.

The stereochemistry of the piperazine group in **10** was demonstrated by 1H NMR spectroscopy. The signal due to H-10 in the artemisinin nucleus at δ 3.95 ppm displays a coupling of 10.4 Hz, corresponding to a trans-diaxial relationship between H-10 and H-9 [52]. Therefore, the piperazine is attached via an α-equatorial bond to C-10 embedded in a chair-like pyran ring. This stereochemistry corresponds to that of all 10-aminoartemisinin derivatives prepared thus far [18,19,20]. However, it was not possible to obtain crystals of **10** suitable for X-ray crystallography, and for this purpose a single crystal of the dimer **16** was used. The structural determination clearly indicated attachment of the piperazine by equatorial bonds from N to C-10 in each of the artemisinin units; the piperazine and the pyran rings of the artemisinins are in the chair conformation (Figure 4 and Supplementary Materials).

### 2.2. N-Glycosylation of DHA-Piperazine ***10***

**Scheme 2:**
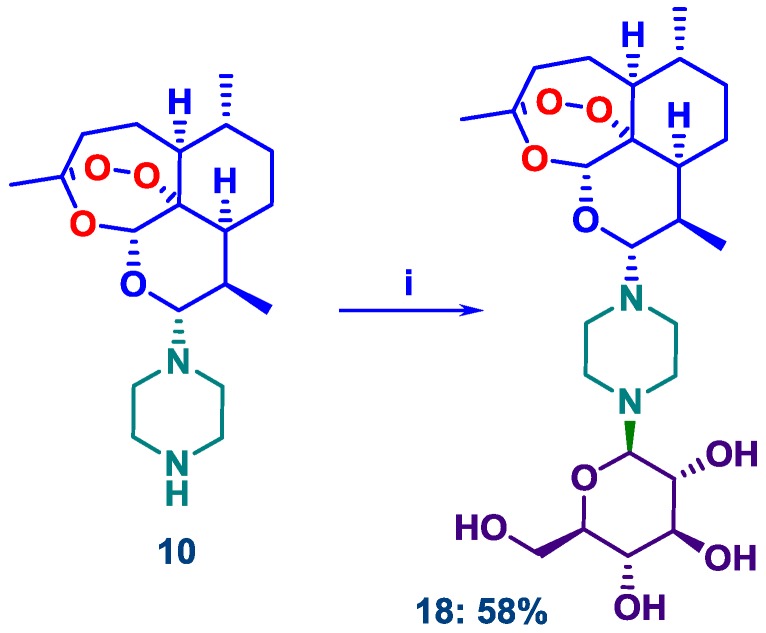
Preparation of the *N*-glucosylated DHA piperazine **18**. i. **10**, D-glucose (3.0 equiv.), 60 °C, MeOH, 24–48 h; 58%.

The Kotchetkov aminoglycosylation reaction [53] involves condensation of amines with aldoses in aqueous alcohol containing inorganic salts [54]. This strikingly effective reaction works well for 2° amines such as indoles with glucose [55] and of relevance to the current case, for substituted piperazines with glucose or galactose [56,57,58]. Whilst attempted *N*-glucosylation of the DHA piperazine **10** with D-glucose by heating in methanol under reflux resulted in some decomposition, by lowering the reaction temperature to 60 °C, formation of the *N*-glucosylated product **18**, a stable white crystalline solid, took place in acceptable yield (Scheme 2).

**Figure 5:**
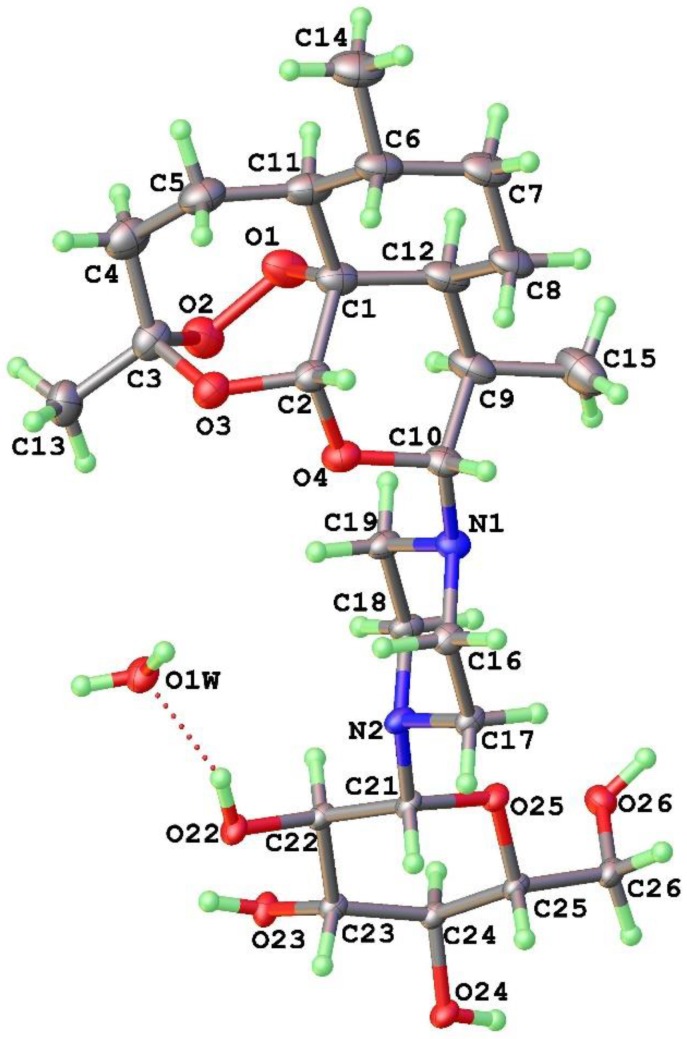
ORTEP plot from X-ray crystallographic structural determination of the *N*-glucosylated DHA piperazine **18** indicating co-crystallization with water, the diaxial arrangement of H-9 and H-10, the chair piperazine and α-equatorial artemisinin C-10 to N-1 bond, and equatorial glucosyl C-21 to N_2_ bond. Numbering is as for the artemisinin nucleus in Figure 1.

In the ^1^H NMR spectrum recorded in methanol-d4, the doublet signal at δ 4.10 ppm with J 10,9 = 10.4 Hz due to H-10 indicates that the equatorial α-configuration of the piperazine linked to the artemisinin nucleus in **10** is retained in **18**. Two multiplets at δ 2.65 and 3.01 ppm are due to the protons on the piperazine linker. The signals at δ 3.19–3.85 ppm correspond to the C-H protons on the glucose moiety. Signals due to the three methyl groups and other protons in the artemisinin nucleus are readily recognized. Details are given in the Experimental Section. The structure was confirmed by X-ray crystallography, wherein crystallization to provide the single crystal incorporated water (Figure 5 and Supplementary Materials). The piperazine ring as for the disubstituted piperazine **16** above is in the chair conformation in which the artemisinin and glucosyl units are attached by equatorial bonds. Although it was initially anticipated that glucose, as a reducing sugar, might affect the artemisinin by reduction, the peroxide remained intact. In any event, the reactions were run without any attempt to exclude air in order to maintain an oxidizing environment.

**Scheme 3:**
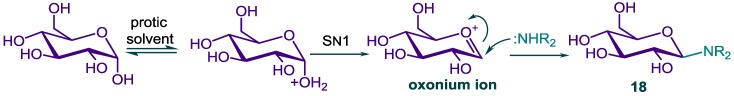
Kochetkov reaction of DHA piperazine **10** with D-glucose via the SN1pathway (NHR_2_ = DHA piperazine **10**).

**Chart 1:**
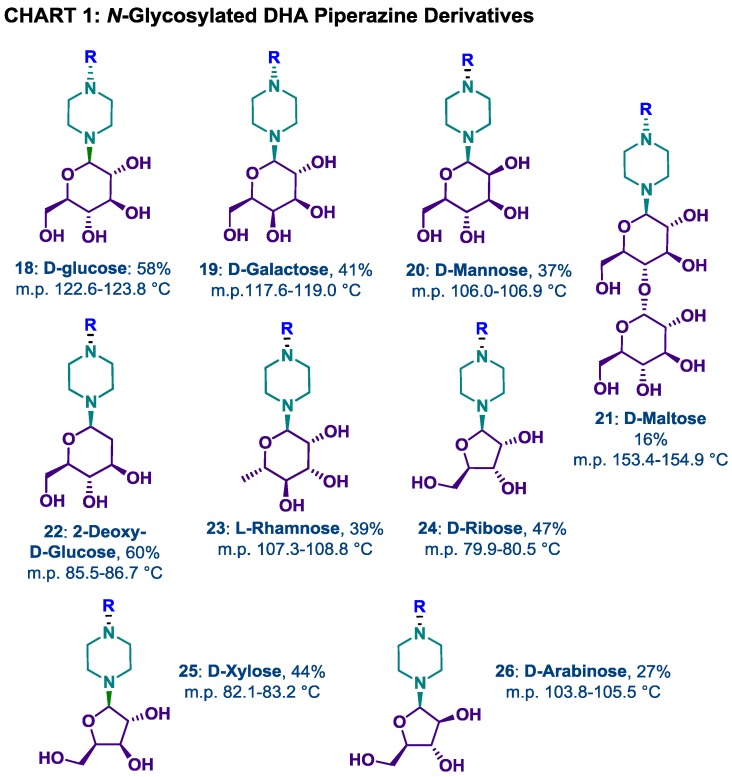
*N*-glycosylated DHA piperazine derivatives prepared by the Kotchetkov reaction from the DHA-piperazine derivative **10** and the corresponding aldose according to Scheme 2 (R = 10-dihydroartemisinyl); isolated yields are given.

The α-configuration of the amine linked to the glucose is assured by the lack of a kinetic anomeric effect exerted on the incoming amine nucleophile in the Kotchetkov reaction. Under general acid catalysis by the solvent, the anomeric hydroxyl group of the glucose is protonated to provide the oxonium intermediate (Scheme 3) that reacts with the DHA piperazine **10** via an SN1 reaction to yield the *N*-glucosylated DHA piperazine **18**. By using this protocol, the other *N*-glycosylated derivatives **19**–**26** were prepared from DHA piperazine **10** and the corresponding aldoses; the sugars used and yields are summarized in Chart 1. Three furanose derivatives **24–26** were also able to be obtained. The disaccharide maltose was also successfully connected to the DHA-piperazine to provide the derivative **21** although in poor yield; this may have been due to the poor solubility of the sugar in methanol. The Kochetkov reaction only worked well for aldoses, in general, no reaction took place for ketose sugars such as fructose and sucrose.

### 2.3. Antimalarial Activities against Different P. falciparum Strains and Cytotoxicities

The *N*-glycosylated DHA piperazine derivatives together with DHA **2**, artemisone **7** and chloroquine as comparator drugs were screened in vitro against chloroquine (CQ)-sensitive *Pf* D10 and CQ-resistant *Pf* W-2 strains [59] by measuring effect on parasite lactate dehydrogenase (pLDH) according to modification of the method of Makler [60]. Antimalarial activities expressed as 50% inhibitory concentrations (IC_50_) [61] are presented in Table 1. Most of the compounds were notably active against both CQ-sensitive and -resistant *Pf*. The galactose **19**, rhamnose **23** and xylose **25** derivatives with IC_50_ values of less than 1 nM were the most active against both strains, with activities comparable to those of artemisone **7** and some 4–5 fold superior to those of DHA (Table 1, column 2).

The derivatives were submitted to comparative cytotoxicity screens on the mouse fibrosarcoma cell line WEHI-164 (Table 1) using the MTT assay as previously described [62]. All showed a selectivity index (SI) with respect to antimalarial activities of >100, indicating that the sugar insertion in the structure did not increase cytotoxicity. Indeed, the mannose and the rhamnose derivatives **20** and **23** had selectivity indices twofold greater than those of DHA or artemisone. Interestingly, the most active compounds against WEHI-164 was the parent DHA piperazine derivative **10** and the xylose derivative **25**. Selected compounds were carried forward for screening against the immortalized myelogenous leukemia, K562, human microvascular endothelial cells (HMEC-1) and fresh human fibroblasts HDF (Table 2) using the MTT assay, as above. [62] Notably, the glucose **18**, maltose **21** and the ribose derivative **24** were the most active against the highly proliferating K562 cell line compared to the endothelial cells or human fibroblasts, although some fourfold less active than artemisone **7** that elicits impressive activity in this screen. All the compounds were significantly less toxic to human fibroblasts than DHA **2** or artemisone **7**. The natural product, camptothecin, a topoisomerase inhibitor and anticancer agent, is more active than artemisone **7**, but also significantly more cytotoxic against HDF (SI < 100).

Overall, the glycosylated piperazine derivatives that are are easily prepared display promising activities against the malaria parasite coupled with very good selectivities. The galactose **19**, rhamnose **23** and xylose **25** piperazine derivatives are the most potent among all of the *N*-glycosylated derivatives. The *enhancement* of antimalarial activities over those of DHA contrasts notably with the *attenuating* effect exerted by attachment of glycosides directly to the hydroxyl group of DHA itself (cf. Figure 3). The trend evidently also supports the contention that C-10 substituted amino-artemisinins are optimum substrates. [22] Thus, further studies evaluating activities of the best compounds in vivo are mandated. Clearly also, given the evidently enhancing effect that the glycoside moiety exerts on aqueous solubilities, we have here a compound class, one or more members of which have the potential to supplant use of artesunate, in particular in the preparation of formulations required for intravenous administration for severe/cerebral malaria. Currently, a dual pack comprising solid artesunate and sterile aqueous sodium bicarbonate that is mixed and diluted with aqueous dextrose immediately prior to injection is used; because artesunate is so easily hydrolyzed to DHA, the emphasis is on immediate injection. The compounds here, like artemisone, are likely to be stable at neutral pH. Thus, results from the next phases of the programme involving target profile evaluation and assessment of pharmacokinetic properties will be reported in due course. Finally, the activities of the *N*-glycosylated piperazine derivatives, and especially of artemisone **7** itself against the myelogenous leukemia cell line K562 are indicative of the viability of the aminoartemisinins at large for further development for antitumour therapy.

## 3. Conclusions

The use of sugars to enhance the selectivity of drugs is currently of great importance. New glycosylated derivatives of artemisinin were conceived on the basis of the incipient concept of carbohydrate-lectin interactions. Appending sugar groups to the artemisinin nucleus should also enhance the properties such as aqueous solubility, and especially render the compounds selectively cytotoxic. Mindful of the problem of attrition of activities observed on converting DHA into *O*-glycosylated DHA derivatives, we focused on preparation of *N*-glycosylated piperazine derivatives that were screened against different strains of *P. falciparum*. The compounds, in contrast to the *O*-glycosylated DHA derivatives, are very active, which vindicates the overall project. Should these compounds turn out to be water soluble, then there is the potential to use these in intravenous formulations for treatment of severe malaria.

## 4. Materials and Methods

### 4.1. General

Dihydroartemisinin was obtained either from the Kunming Pharmaceutical Corporation, Kunming, China; or from Haphacen, Hanoi College of Pharmacy, Vietnam. Other chemicals were purchased from commercial sources and used without further purification. Methanol (AR grade) was used as received. Dichloromethane was dried and distilled from calcium hydride. Toluene was dried over sodium and distilled from sodium benzophenone ketyl prior to use. Ethyl acetate and hexane for column chromatography were distilled from calcium chloride. Triethylamine was dried over calcium hydride and stored over sodium hydroxide pellets. TLC was performed with Merck (Darmstadt, Germany) Kieselgel 60 F254 plates and visualized either with ultra violet light (254 nm) or by heating after treatment with ammonium molydbate in 10% concentrated sulfuric acid. Column chromatography was performed with Merck silica gel 60 (0.04–0.063 mm, Darmstadt, Germany).

Melting points were recorded on a Leica (Wetzlar, Germany) Microscope Heating Stage 350 and are corrected. ^1^H and ^13^C NMR spectral data, unless otherwise stated, were obtained from samples in CDCl_3_ or CD_3_OD. ^1^H NMR spectrum was recorded on Bruker-400 spectrometer (Bruker AG, Karlsruhe, Germany) operating at 400 MHz and ^13^C NMR spectrum was recorded on Bruker-300 spectrometer (Billerica, MA, USA) operating at 75.4 MHz for ^13^C. Chemical shifts were reported in ppm relative to internal standard tetramethylsilane (0.3% v/v) as 0.0 ppm for ^1^H and CDCl_3_ as 77.0 ppm for ^13^C. Coupling constant was recorded in Hz. Abbreviations: s, singlet; d, doublet; t, triplet; q, quartet; m, multiplet. Infrared spectra were recorded either on a Perkin (Peterborough, UK) Elmer Spectrum One spectrometer. Single crystal X-ray structure measurements were carried out on a Bruker (Billerica, MA, USA). Smart-APEX CCD four-circle diffractometer. All computations in the structure determination and refinement were performed on Silicon Graphics Indy computer using programs of the Siemens SHELXTL PLUS (version 5) (Munich, Germany) package (see Supplementary Materials).

### 4.2. Synthesis of Glycoside Derivatives: 10α-(1-Piperazino)-10-deoxo-10-dihydroartemisinin 10 and (bis-11′,4-piperazino)-10α-deoxo-10-dihydroartemisinin ***16***

Dimethyl sulfoxide (50 µL, 0.704 mmol) and then oxalyl chloride (0.4 mL, 4.73 mmol) were added to a suspension of dihydroartemisinin **2** (2.0 g, 7.04 mmol) in toluene (10 mL) under nitrogen which was then stirred for 30 min, during which time the DHA dissolved to form a clear amber solution. This was then added via cannula to a solution of piperazine (1.8 g, 21.21 mmol) in dichloromethane (20 mL). The resulting mixture was stirred for another 12 h at ambient temperature, and then diluted with more dichloromethane (40 mL). This was then filtered through a pad of Celite, and the filtrate was washed with water (3 × 30 mL) and brine (30 mL). The organic layer was dried (MgSO_4_) and filtered, and the filtrate was evaporated under reduced pressure to leave a residue that was submitted to chromatography over silica gel. Elution with dichloromethane-methanol-triethylamine (10:1:0.1) gave the product **10** as a cream white solid (1.8 g, 74%), m.p. 99–100 °C, [α]_D_^22^ + 12.58° (*c* 0.54, CHCl_3_). 1H NMR δ 0.81 (d, *J =* 7.0 Hz, 3H, H-14), 0.95 (d, *J* = 6.2 Hz, 3H, H-15), 1.16–1.35 (m, 2 H), 1.39 (s, 3H, H-13), 1.42–1.58 (m, 3H), 1.59-1.74 (m, 3H), 1.83–1.92 (m, 1 H), 1.96–.2.03 (m, 1H), 2.29–2.42 (m, 1H), 2.54–2.65 (m, 4H, piperazine), 2.79–2.89 (m, 4H, piperazine), 2.93 (s, 1H, NH), 2.96–3.0 (m, 1H), 3.97 (d, *J* = 10.3 Hz, 1H, H-10), 5.27 (s, 1H, H-12); IR (film) ν_max_ 3327, 2927, 2872, 2346, 1714, 1645, 1450, 1376, 1319, 1260, 1198, 1161, 1056, 982, 928, 880, 852, 804, 733, 664 cm^−1^. MS (CI, CH4) *m*/*z* (%) 221.1 (8), 260.1 (11), 383.2 (12), 412.3 (3), 427.3 (22), 441.3 (100), 442.3 (25), 469.3 (19), 487.3 (8). HRMS (ESI): *m*/*z* calcd. for C_19_H_33_N_2_O_4_ 353.2440 [M + H]^+^, found 353.2482. 

Dimethyl sulfoxide (37.5 µL, 0.1 equiv) was added into a stirred suspension of DHA (1.5 g, 5.275 mmol) in toluene (15 mL) at room temperature under nitrogen. Oxalyl chloride (0.53 mL, 1.15 equiv) was slowly added into the suspension which during the course of the addition became a pale amber solution. This was stirred for 1 h, and then treated dropwise with a solution of piperazine (0.45 g, 1 equiv.) in dichloromethane (10 mL); the resulting mixture was stirred overnight. This was then quenched with saturated aqueous NaHCO_3_ (40 mL), and extracted with ethyl acetate (4 × 30 mL). The organic layers were combined, and the combined layer was washed with brine (40 mL) and dried (MgSO_4_). After filtration, the filtrate was evaporated under reduced pressure to leave a residue that was submitted to chromatography over silica gel. Elution with hexane-ethyl acetate 4:1 gave the product **16** (568 mg, 35%) as a white crystalline solid. Crystallization from ethyl acetate-hexane gave colourless plates, m.p. 149–150 °C, [α]_D_^22^ 2.31° (*c* 0.58, CHCl_3_). ^1^H NMR δ 0.79 (d, *J* = 7.0 Hz, 6H, 14-H), 0.94 (d, *J* = 5.9 Hz, 6H, H-15), 1.21–1.34 (m, 6H), 1.40 (s, 6H, H-13), 1.45–1.57 (m, 4H), 1.58–1.60 (m, 4 H), 1.64–1.76 (m, 4 H), 1.83–1.91 (m, 2 H), 1.94–2.04 (m, 2 H), 2.29–2.41 (m, 2 H), 2.58–2.60 (m, 4H, piperazine), 2.99–3.02 (m, 4 H, piperazine), 3.99 (d, *J* = 10 Hz, 2H, H-10,), 5.27 (s, 2H, H-12). IR (film) ν_max_ 2927, 2871, 1651, 1522, 1454, 1376, 1327, 1304, 1279, 1252, 1226, 1207, 1180, 1163, 1130, 1101, 1056, 1041, 1024, 979, 943, 926, 880, 847, 827, 734 cm^–1^. MS (CI, CH4) *m*/*z* (%) 221.1 (4), 306.2 (8), 346.2 (4), 455.3 (3), 469.3 (14), 513.4 (36), 527.4 (100), 530.5 (11), 557.5 (3), 573.4 (2). HRMS (ESI) *m*/*z*: calcd for C_34_H_55_N_2_O_8_ 619.3958 [M + H] ^+^, found 619.3929; Calcd. for C_34_H_54_N_2_O_8_ C 65.99, H 8.80, N 4.52; found C 66.37, H 8.93, N 4.18%.

### 4.3. Synthesis of Glycoside Derivatives

#### 4.3.1. D-Glucose

10α-(Piperazinyl)-10-deoxyartemisinin **10** (654 mg, 1.85 mmol) was stirred with D-glucose (1.0 g, 5.55 mmol, 3.0 equiv.) in methanol (20 mL) at 60 °C for 24 h; an open system was used, that is, no precautions were taken to exclude air. The solvent was then evaporated under reduced pressure, and the residue was dissolved in ethyl acetate (40 mL). The solution was washed with saturated aqueous NaHCO_3_ (3 × 25 mL), and then brine (25 mL). The organic layer was separated and dried (MgSO_4_), and filtered. The filtrate was evaporated under reduced pressure, and the residue was submitted to chromatography on silica gel. Elution with ethyl acetate-methanol (20:1) gave the glucose derivative **18** (549 mg, 58%). The product was recrystallized from dichloromethane/ hexane to afford **18** as fine white needles, m.p. 122.6–123.8 °C. ^1^H NMR (400 MHz, CD_3_OD): δ 0.81 (d, *J* = 7.2 Hz, 3H, H-14), 0.99 (d, *J* = 6.4 Hz, 3H, H-15), 0.94–1.57 (m, 3H), 1.37 (s, 3H, H-13), 1.40–1.95 (m, 7H), 2.22–2.32 (m, 1H), 2.46–2.64 (m, 8H), 2.63–2.68 (m, 1H), 2.88–3.54 (m, 7H), 3.6–3.63 (m, 2H), 3.99 (d, *J* = 10.0 Hz, 1H, H-10), 4.43–4.49 (m, 2H), 5.41 (s, 1H, H-12) ppm. ^13^C NMR (75.4 MHz, CDCl_3_): δ 13.4, 20.2, 21.6, 24.6, 25.9, 28.5, 34.2, 36.3, 37.2, 45.8, 46.9, 47.5, 51.7, 62.1, 68.9, 70.4, 77.5, 80.2, 90.5, 91.6, 94.2, 103.8 ppm. IR (film) ν_max_ = 551, 617, 832, 879, 924, 941, 982, 1020, 1039, 1058, 1075, 1099, 1115, 1130, 1208, 1277, 1299, 1328, 1379, 1455, 1648, 2868, 2924, 3417 cm^–1^; HRMS (ESI) calcd. for C_25_H_43_N_2_O_9_ 515.2969 [M + H]^+^, found 515.2976.

#### 4.3.2. D-Galactose

A solution of 10α-(piperazinyl)-10-deoxyartemisinin **10** (498 mg, 1.41 mmol) and D-galactose (760 mg, 4.2 mmol) in methanol (15 mL) at 60 °C was stirred for 36 h. After workup according to the foregoing procedure, the crude product was submitted to chromatography with dichloromethane-methanol (15:1) to afford the galactose derivative **19** (294 mg, 41%) as a white microcrystalline solid, m.p. 117.6–119.0 °C. ^1^H NMR (400 MHz, CDCl_3_): δ 0.69 (d, *J* = 8.0 Hz, 3H, H-14), 0.87 (d, *J* = 6.4 Hz, 3H, H-15), 0.94–1.57 (m, 3H), 1.25 (s, 3H, H-13), 1.40–2.20 (m, 8H), 2.38–2.51 (m, 7H), 2.46–2.64 (m, 8H), 2.79–2.83 (m, 4H), 2.88–3.54 (m, 7H), 3.12–3.87 (m, 7H), 3.99 (d, *J* = 10.0 Hz, 1H, H-10), 4.43–4.49 (m, 2H), 5.29 (s, 1H, H-12) ppm. ^13^C NMR (75.4 MHz, CDCl_3_): δ 11.7, 14.1, 15.3, 20.6, 21.0, 22.3, 25.4, 26.6, 27.6, 28.2, 29.0, 29.2, 30.4, 32.2, 34.9, 35.8, 37.0, 38.1, 41.0, 41.9, 45.7, 46.4, 47.7, 48.7, 52.4, 52.5, 53.1, 57.2, 62.3, 63.2, 67.6, 70.2, 75.5, 76.5, 80.9, 81.0, 91.2, 91.6, 92.3, 95.7, 104.7 ppm. IR (film) ν_max_ = 510, 551, 660, 693, 746, 766, 834, 851, 880, 924, 940, 957, 981, 10101, 1061, 1101, 1134, 1177, 1208, 1279, 1383, 1419, 1455, 1633, 1706, 2870, 2922, 3395 cm^−1^; HRMS (ESI): calcd. For C_25_H_43_N_2_O_9_ 515.2969 [M + H]^+^, found 515.2987.

#### 4.3.3. D-Mannose

A solution of 10α-(piperazinyl)-10-deoxyartemisinin **10** (629 mg, 1.78 mmol) and D-mannose (960 mg, 5.34 mmol) in methanol (20 mL) at 60 °C was stirred for 36 h. After workup according to the procedure in i. above, the crude product was submitted to chromatography with dichloromethane-methanol (15:1) to afford the mannose derivative **20** (340 mg, 37%) as a fine white powder, m.p. 106.0–106.9 °C. ^1^H NMR (400 MHz, CDCl_3_) δ 0.79 (d, *J* = 7.2 Hz, 3H, H-14), 0.97 (d, *J* = 7.6 Hz, 3H, H-15), 0.83–1.38 (m, 6H), 1.42 (s, 3H, H-13), 1.44–2.16 (m, 7H), 2.30–3.08 (m, 12H), 3.50 (d, *J* = 9.2 Hz, 1H, H-10), 3.65–4.01 (m, 7H), 5.28 (s, 1H, H-12) ppm. ^13^C NMR (75.4 MHz, CDCl_3_) δ 13.5, 14.2, 20.4, 21.7, 22.7, 24.8, 26.2, 28.6, 31.0, 31.7, 34.4, 36.4, 37.5, 45.9, 47.1, 51.8, 54.7, 61.3, 62.7, 69.4, 70.99, 71.7, 75.6, 80.1, 80.4, 83.7, 90.6, 91.8, 96.7, 102.0, 104.0 ppm. IR (film) ν_max_ = 572, 642, 658, 734, 756, 803, 826, 879, 927, 982, 1040, 1380, 1448, 1631, 1708, 2929, 3433 cm^−1^; HRMS (ESI): calcd. for C_25_H_43_N_2_O_9_ 515.2969 [M + H]^+^, found 515.2980.

#### 4.3.4. D-Maltose

According to the general procedure, the reaction was started with 10α-(piperazinyl)-10-deoxyartemisinin **10** (421 mg, 1.19 mmol) and maltose (1.2 g, 3.6 mmol) in methanol (20 mL) at 60 °C for 48 h. The solvent was removed directly from the reaction mixture by evaporation under reduced pressure, and the residue was submitted directly to column chromatography. Elution with dichloromethane-methanol (4:1) gave the maltose derivative **21** (131 mg, 16%), that slowly precipitated as a fine white microcrystalline powder from ethyl acetate-methanol (5:1), m.p. 153.4–154.9 °C. ^1^H NMR (400 MHz, CD_3_OD): δ 0.70 (d, *J* = 6.4 Hz, 3H, H-14), 0.92 (d, *J* = 6.0 Hz, 3H, H-15), 0.91–1.67 (m, 7H), 1.45 (s, 3H, H-13), 1.84–2.33 (m, 8H), 2.90–4.35 (m, 14H), 4.01 (d, J = 10.2 Hz, 1H, H-10), 5.64 (s, 1H, H-12) ppm. ^13^C NMR (75.4 MHz, CD_3_OD): δ 13.5, 21.7, 26.4, 27.5, 29.9, 31.1, 32.6, 35.3, 38.4, 40.4, 42.0, 46.8, 52.7, 57.3, 61.1, 63.2, 63.5, 68.5, 75.7, 77.7, 79.2, 80.3, 85.2, 90.8, 91.7, 96.0, 100.9, 102.3, 173.4 ppm. IR (film) ν_max_ = 824, 836, 879, 925, 989, 1015, 1054, 1112, 1131, 1147, 1178, 1210, 1277, 1305, 1328, 1383, 1455, 1640, 2868, 2924, 2949, 3392 cm^−1^; HRMS (ESI) calcd. for C_31_H_53_N_2_O_14_ 677.3497 [M + H]^+^, found 677.3475.

#### 4.3.5. 2-Deoxy-D-glucose

A solution of 10α-(piperazinyl)-10-deoxyartemisinin **10** (447 mg, 1.27 mmol) and 2-deoxy-D-glucose (625 mg, 3.81 mmol) in methanol (15 mL) at 60 °C was stirred for 24 h. After workup according to the procedure in i. above, the crude product was submitted to chromatography with dichloromethane-methanol (20:1) to afford the 2-deoxyglucose derivative **22** (377 mg, 60%) as a fine white powder, m.p. 85.0–86.7 °C. ^1^H NMR (400 MHz, CDCl_3_): δ 0.80 (d, *J* = 6.8 Hz, 3H, H-14), 0.95 (d, *J* = 5.6 Hz, 3H, H-15), 0.85–1.05 (m, 1H), 1.38 (s, 3H, H-13), 1.23–1.56 (m, 7H), 1.68–2.67 (m, 12H), 2.91–4.03 (m, 8H), 5.27 (s, 1H, H-12) ppm. ^13^C NMR (75.4 MHz, CDCl_3_): δ 13.5, 20.4, 21.7, 24.8, 26.0, 28.7, 29.8, 35.7, 36.4, 37.5, 45.9, 47.0, 48.2, 62.4, 72.6, 72.7, 77.6, 80.4, 90.6, 91.4, 91.7, 104.1 ppm. IR (film) v_max_ = 825, 879, 984, 1054, 1208, 1378, 1449, 1632, 1708, 2871, 2927, 3414 cm^−1^; HRMS (ESI) calcd. for C_25_H_43_N_2_O_8_ 499.3019 [M+H]^+^, found 499.3012.

#### 4.3.6. L-Rhamnose

A solution of 10α-(piperazinyl)-10-deoxyartemisinin **10** (337 mg, 0.96 mmol) and L-rhamnose (473 mg, 2.88 mmol) in methanol (10 mL) at 60 °C was stirred for 24 h. After workup according to the procedure in i. above, the crude product was submitted to chromatography with dichloromethane-methanol (15:1) to afford the rhamnose derivative **23** (185 mg, 38.5%) that slowly precipitated from dichloromethane-hexane as a white powder, m.p. 107.3–108.8 °C. ^1^H NMR (400 MHz, CDCl_3_): δ 0.81 (d, *J* = 6.8 Hz, 3H, H-14), 0.98 (d, *J* = 6.0 Hz, 3H, H-15), 1.04–1.29 (m, 7H), 1.36 (s, 3H, H-13), 1.42–2.49 (m, 15H), 2.85–4.80 (m, 11H), 4.01 (d, 1H, *J* = 10.2 Hz, H-10), 5.41 (s, 1H, H-12) ppm. ^13^C NMR (75.4 MHz, CDCl_3_): δ 13.6, 17.8, 20.4, 21.8, 24.9, 26.1, 28.7, 34.4, 36.5, 37.5, 46.0, 46.7, 48.8, 49.3, 51.9, 69.0, 73.1, 74.0, 75.2, 80.4, 90.6, 91.8, 93.0, 104.1 ppm. IR (film) ν_max_ = 826, 879, 925, 982, 1015, 1058, 1208, 1280, 1277, 1454, 1633, 2870, 2927, 3416 cm^−1^; HRMS (ESI), calcd. for C_25_H_43_N_2_O_8_499.3019, *m*/*z* [M + H]^+^ found 499.3002. 

#### 4.3.7. D-Ribose

A solution of 10α-(piperazinyl)-10-deoxyartemisinin **10** (377 mg, 1.07 mmol) and D-ribose (480 mg, 3.21 mmol) in methanol (10 mL) at 60 °C was stirred for 24 h. After workup according to the procedure in i. above, the crude product was submitted to chromatography with dichloromethane-methanol (20:1) to afford the ribose derivative **24** (247 mg, 47%) that slowly precipitated from dichloromethane-hexane as a white powder, m.p. 79.9–80.5 °C. ^1^H NMR (400 MHz, CDCl_3_): δ 0.78 (d, *J* = 7.2 Hz, 3H, H-14), 0.95 (d, *J* = 6.0 Hz, 3H, H-15), 0.83–1.38 (m, 5H), 1.41 (s, 3H, H-13), 1.44–2.02 (m, 7H), 2.21–2.98 (m, 12H), 3.32–4.41 (m, 9H), 5.28 (s, 1H, H-12) ppm. ^13^C NMR (75.4 MHz, CDCl_3_): δ 13.5, 20.4, 21.7, 24.8, 26.1, 28.6, 34.4, 36.4, 37.5, 45.9, 51.8, 54.4, 54.7, 62.1, 69.9, 71.5, 72.7, 73.8, 80.4, 80.4, 90.5, 91.7, 91.8, 100.4, 104.0, 104.1 ppm. IR (film) ν_max_ = 617, 826, 858, 879, 926, 1041, 1130, 1207, 1280, 1378, 1454, 1632, 1720, 2871, 2927, 3424 cm^−1^; HRMS (ESI): calcd. for C_24_H_41_N_2_O_8_ 485.2863 [M + H]^+^, found 485.2832.

#### 4.3.8. D-Xylose

A solution of 10α-(piperazinyl)-10-deoxyartemisinin **10** (421 mg, 1.19 mmol) and D-xylose (536 mg, 3.57 mmol) in methanol (10 mL) at 60 °C was stirred for 24 h. After workup according to the procedure in i. above, the crude product was submitted to chromatography with dichloromethane-methanol (15:1) to afford the xylose derivative **25** (258 mg, 44%), that slowly precipitated from dichloromethane-hexane as a white powder, m.p. 82.1–83.2 °C. ^1^H NMR (400 MHz, CDCl_3_): δ 0.78 (d, *J* = 6.8 Hz, 3H, H-14), 0.94 (d, *J* = 6.4 Hz, 3H, H-15), 0.79–1.07 (m, 2H), 1.41 (s, 3H, H-13), 1.18–1.55 (m, 6H), 1.63–2.05 (m, 4H), 2.30–2.38 (td, *J* = 14.4, 3.6 Hz, 1H), 2.54–2.66 (m, 5H), 2.91–3.97 (m, 12H), 3.78 (d, *J* = 10.4 Hz, 1H, H-10), 5.27 (s, 1H, H-12) ppm. ^13^C NMR (75.4 MHz, CDCl_3_): δ 13.5, 20.4, 21.8, 24.8, 26.1, 28.6, 28.7, 34.4, 36.5, 37.5, 45.8, 46.0, 47.3, 47.9, 51.9, 67.7, 69.0, 70.0, 78.2, 80.3, 80.4, 90.8, 91.8, 95.3, 104.1, 104.2 ppm. IR (film) ν_max_ = 826, 879, 925, 979, 1040, 1129, 1207, 1281, 1378, 1455, 1634, 1722, 2871, 2928, 3420 cm^−1^; HRMS (ESI): calcd. for C_24_H_41_N_2_O_8_ 485.2863 [M + H]^+^, found 485.2817.

#### 4.3.9. D-Arabinose

A solution of 10α-(piperazinyl)-10-deoxyartemisinin **10** (314 mg, 0.80 mmol) and D-arabinose (400 mg, 2.67 mmol) in methanol (10 mL) at 60 °C was stirred for 24 h. After workup according to the procedure in i. above, the crude product was submitted to chromatography with dichloromethane-methanol (15:1) to afford the arabinose derivative **26** (114 mg, 27%), that slowly precipitated from dichloromethane-hexane as a white powder, m.p. 103.8–105.5 °C. ^1^H NMR (400 MHz, CDCl_3_): δ 0.79 (d, *J* = 6.8 Hz, 3H, H-14), 0.94 (d, *J* = 6.0 Hz, 3H, H-15), 0.79–1.07 (m, 1H), 1.40 (s, 3H, H-13), 1.13–2.24 (m, 9H), 2.30–3.00 (m, 11H), 3.49–4.16 (m, 8H), 5.28 (s, 1H, H-12) ppm. ^13^C NMR (75.4 MHz, CDCl_3_): δ 13.5, 20.4, 21.7, 24.8, 26.1, 28.6, 31.0, 34.4, 36.4, 37.4, 45.8, 47.3, 48.0, 51.8, 54.4, 54.7, 62.1, 67.2, 68.2, 69.8, 71.5, 73.8, 74.6, 80.4, 90.5, 91.7, 95.4, 100.5, 104.0, 104.4 ppm. IR (film) ν_max_ = 825, 879, 925, 978, 1053, 1085, 1131, 1206, 1378, 1454, 1632, 1715, 2870, 2926, 3424 cm^−1^; HRMS (ESI): calcd. for C_24_H_41_N_2_O_8_ 485.2863 [M + H]^+^, found 485.2817.

### 4.4. Biological Assays

All the reagents unless indicated otherwise were purchased from Sigma (Sigma Italia, Milan, Italy).

#### 4.4.1. Cultures of Pf and In Vitro Antimalarial Assays

The *Pf* strains D10 (CQ-sensitive), and W-2 (CQ-resistant) were cultured in vitro as described by Trager and Jensen with minor modifications [60]. Parasites were maintained at 5% hematocrit (human type A-positive red blood cells) in RPMI 1640 (EuroClone, Celbio) medium with the addition of 10% heat inactivate human serum, 20 mM Hepes and 2 mM glutamine (Euroclone). The cultures were maintained at 37 °C in a standard gas mixture consisting of 1–3% O_2_, 5% CO_2_, and 92–94% N_2_. Compounds were dissolved in either water or DMSO and then diluted with medium to achieve the required concentrations (final DMSO concentration <1%, which is non-toxic to the parasite). Asynchronous cultures of *Pf* with parasitaemia of 1–1.5% and 1% final hematocrit were aliquoted into 96-well flat-bottom microplates (COSTAR) with serial dilutions of test compounds and incubated for 72 h at 37 °C. Parasite growth was determined spectrophotometrically (OD650) by measuring the activity of the parasite lactate dehydrogenase (pLDH) according to a modification of the method of Makler in control and drug-treated cultures [61]. The antimalarial activity is expressed as 50% inhibitory concentrations (IC_50_); each IC_50_ value is the mean and standard deviation of at least three separate experiments performed in duplicate [62].

#### 4.4.2. Cell Cytotoxicity Assays

A long term microvascular endothelial cell line (HMEC-1) immortalized by SV 40 large T antigen was kindly provided by Dr. Francisco J. Candal, Center for Disease Control, Atlanta, GA, USA. Cells were maintained in MCDB 131 medium (Invitrogen, Milan, Italy) supplemented with 10% fetal calf serum (HyClone, Celbio, Milan, Italy), 10 ng/mL of epidermal growth factor (Chemicon), 1 µg/mL of hydrocortisone, 2 mM glutamine, 100 units/ml of penicillin, 100 µg/mL of streptomycin and 20 mM Hepes buffer (EuroClone). K562, the human myelogenous leukemia cell line and WEHI 164 murine fibrosarcoma line were cultured in RPMI 1640 supplemented with 2 mM glutamine, 100 units/ml of penicillin, 100 µg/mL of streptomycin and 10% fetal calf serum. Fibroblasts from skin biopsies were maintained in DMEM (EuroClone, Pero, Italy) supplemented with 10% fetal calf serum (HyClone, Logan, UT, USA), 2 mM glutamine, 100 U/mL of penicillin and 100 µg/mL of streptomycin.

For the cytotoxicity assays, HMEC-1 and WEHI 164 cells were seeded in 96 well flat bottom tissue culture clusters at 10^4^ cells/well; HDF were seeded at 1.5 × 10^4^ cells/well and let to adhere for 24 h before the drug treatment; K562 were seeded in round bottom clusters at 1.5 × 10^4^ cells/well. Cells were then treated with serial dilutions of test compounds and cell proliferation evaluated using the MTT assay as previously described [62]. Plates were incubated for 72 h at 37 °C in 5% CO_2_, then 20 µL of a 5 mg/ml solution of 3-(4,5-dimethylthiazol-2-yl)-2,5-diphenyltetrazolium bromide (MTT) in PBS were added for an additional 3 h at 37 °C. The plates were then centrifuged, the supernatants discarded and the dark blue formazan crystals dissolved using 100 µL of lysing buffer consisting of 20% (w/v) of a solution of SDS (sodium dodecyl sulfate), 40% of *N*,*N*-dimethyl formamide (Merck) in distilled water adjusted with 80% acetic acid to pH 4.7. The plates were then read on a microplate reader (Molecular Devices Co., Menlo Park, CA, USA) at a test wavelength of 550 nm and a reference wavelength of 650 nm. The results are expressed as IC_50_ which is the dose of compound necessary to inhibit cell growth by 50%. All the tests were performed in triplicate at least three times.

#### 4.4.3. IC_50_ Calculation

The results of the antimalarial and cytotoxicity assays were expressed as the percent viability compared to the untreated controls, calculated with the following formula: 100([OD of treated sample blank]/[OD of untreated sample blank]) (OD, optical density). As a blank, uninfected RBCs were used for the antimalarial assays or media without cells for the cytotoxicity assays. The percent viability was plotted as a function of drug concentrations, and the curve fitting was obtained by nonlinear regression analysis using a four parameter logistic method (software Gen5 1.10 provided with the Synergy4 plate reader [Biotek]). The IC_50_ was extrapolated as the dose that induced a 50% inhibition of parasites or cell viability.

## Figures and Tables

**Table 1:** In vitro screening of the *N*-glycosylated DHA piperazine derivatives against *P. falciparum* CQ-sensitive D10 and CQ-resistant W2 strains, and cytotoxicity on the mouse fibroblast cell line WEHI-164.

Compound	D10 (CQ-S) Mean ± S.D. IC_50_, nM ^a^	Ratio IC_50_ cpd/IC_50_ DHA	SI IC_50_WEHI-164/IC_50_ cpd ^b^	W-2 (CQ-R) Mean ± S.D. IC_50_, nM ^a^	Ratio IC_50_ cpd/IC_50_ DHA	SI IC_50_WEHI-164/IC_50_ cpd ^b^	WEHI-164 Mean ± S.D. IC_50_, nM ^c^
**10**	1.4 ± 0.5	0.41	107	1.1 ± 0.4	0.73	136	150 ± 10
**18**	1.3 ± 0.1	0.38	215	1.2 ± 0.3	0.8	233	280 ± 80
**19**	0.87 ± 0.25	0.26	195	0.71 ± 0.2	0.5	239	170 ± 30
**20**	5.7 ± 2.9	1.7	596	4.4 ± 2.3	2.9	772	3,400 ± 400
**21**	1.9 ± 1.1	0.56	131	1.5 ± 0.5	1.0	166	250 ± 90
**22**	2.3 ± 1.0	0.68	222	1.1 ± 0.4	0.73	463	510 ± 100
**23**	0.74 ± 0.34	0.22	878	0.74 ± 0.3	0.49	878	650 ± 60
**24**	4.8 ± 2.9	1.4	102	4.1 ± 3.2	2.7	119	490 ± 200
**25**	0.66 ± 0.2	0.19	242	0.58 ± 0.3	0.39	275	160 ± 40
**26**	1.3 ± 0.8	0.38	161	0.82 ± 0.25	0.55	256	210 ± 10
DHA **2**	3.4 ± 1.6		211	1.5 ± 0.3		480	720 ± 140
Artemisone **7**	1.1 ± 0.4	0.32	269	0.9 ± 0.4	0.6	328	296 ± 59
Chloroquine	23.4 ± 10.1			263.6 ± 142.9			>10,000

^a^ Results are expressed as mean IC_50_ ± SD of at least three different experiments each performed in duplicate or triplicate; ^b^ Selectivity Index SI: IC_50_ WEHI 164/. IC_50_ for respective compounds against each strain of *P. falciparum*; ^c^ Results are expressed as mean IC_50_ ± SD of at least three 72 h experiments, each performed in duplicate or triplicate.

**Table 2:** Cytotoxicities of *N*-glycosylated DHA piperazine derivatives against the myelogenous leukemia cell line K562, human dermal endothelial cells HMEC-1, and fresh human dermal fibroblasts HDF.

Compound	Mean (±S.D.) IC_50_, µM ^a^		
	K562b	HMEC ^b^	HDF ^b^
**18**	0.81 ± 0.24	4.41 ± 2.29	382.5 ± 31.2
**21**	0.78 ± 0.15	2.50 ± 0.80	533.6 ± 9.9
**23**	1.95 ± 0.48	nd	313.6 ± 85.6
**24**	0.87 ± 0.05	2.39 ± 1.51	389.1 ± 8.6
DHA **2**	2.76 ± 0.66	1.16 ± 0.40	88.7 ± 13.3
Artemisone **7**	0.26 ± 0.04	nd	88.3 ± 33.8
Camptothecin	0.03 ± 0.01	nd	2.62 ± 0.84

^a^ Results are expressed as mean IC_50_ ± SD of at least three different experiments each performed in duplicate or triplicate; ^b^ MTT assay conducted for 72 h.

## Supplementary Materials

The Supplementary Materials are available online S1: HRMS spectra of *N*-glycosylated derivatives **18**–**26**, Tables S1–S6: X-Ray structural tables for compound **16**; Tables S7–S13: X-Ray structural tables for compound **18**.
