# Supplementary material for: Facile Preparation of N-Glycosylated 10-Piperazinyl Artemisinin Derivatives and Evaluation of Their Antimalarial and Cytotoxic Activities
Source: Molecules. 2018 Jul 13;23(7):1713. doi: 10.3390/molecules23071713 (PMC6100044; doi:10.3390/molecules23071713)
Supplement: Supplementary file 1 [file molecules-23-01713-s001.pdf]

Facile Preparation of *N*-Glycosylated 10-Piperazinyl Artemisinin Derivatives and Evaluation of their Antimalarial and Cytotoxic Activities

Yuet Wu[1*], Silvia Parapini[2,5*], Ian D. Williams[1], Paola Misiano[3], Ho Ning Wong[4], Donatella Taramelli[3,5], Nicoletta Basilico[2,5**] and Richard K Haynes[1,4**]

1. Department of Chemistry, The Hong Kong University of Science and Technology, Clear Water Bay, Kowloon, Hong Kong
2. Department of Biomedical, Surgical and Dental Sciences (DiSBIOC), University of Milan, Via Pascal 36, 20133 Milan, Italy
3. Department of Pharmacological & Biomolecular Sciences (DiSFeB), University of Milan, Via Pascal 36, 20133 Milan, Italy
4. Center of Excellence for Pharmaceutical Sciences, Faculty of Health Sciences, North-West University, Potchefstroom, South Africa.
5. Inter University Center for Malaria Research, Italian Malaria Network

* Contributed equally to the manuscript.

**Corresponding authors: Nicoletta Basilico (biology) and Richard K. Haynes (chemistry).

Supplementary Material:

Figure S1- HRMS spectra of *N*-glycosylated derivatives 18-26.

i. D-glucose:

Chemical Formula: $C_{25}H_{42}N_2O_9$
Exact Mass: 514.2890

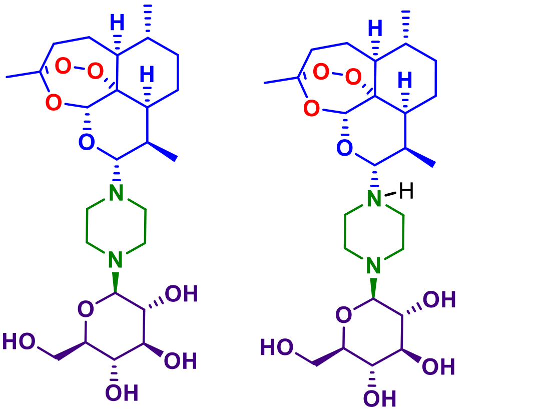

18

Chemical Formula: $C_{25}H_{43}N_2O_9$
Exact Mass: 515.2969

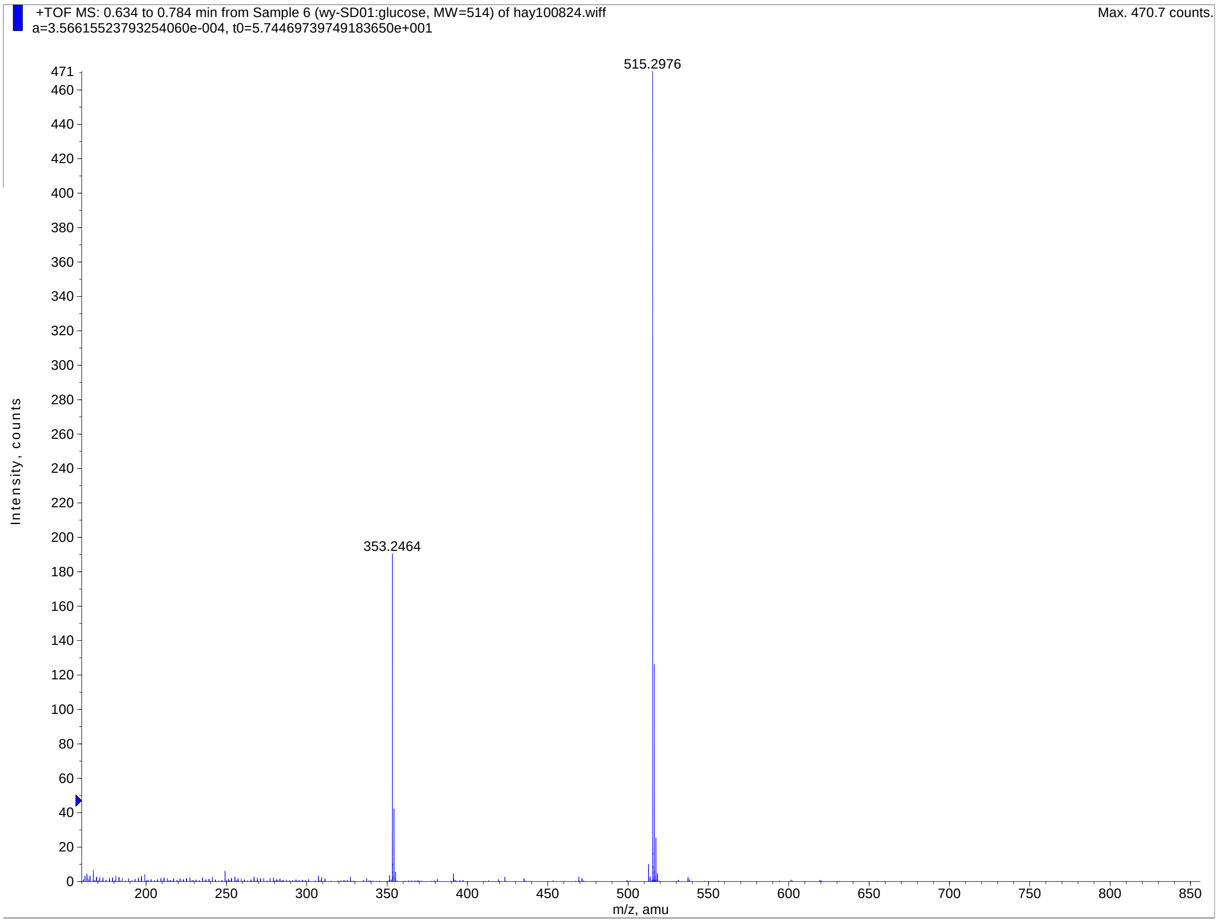

HRMS (ESI) calcd for $C_{25}H_{42}N_2O_9H$ 515.2969 $[M+H]^+$, found 515.2976.

ii. D-Galactose:

Chemical Formula: $C_{25}H_{42}N_2O_9$
Exact Mass: 514.2890

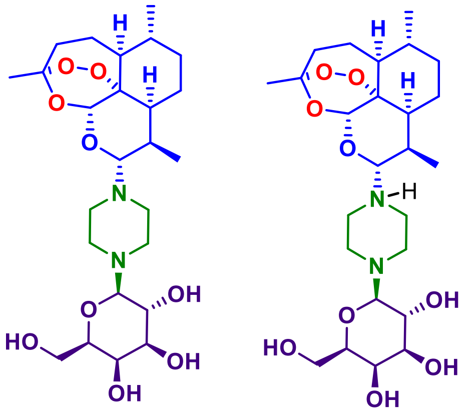

19

Chemical Formula: $C_{25}H_{43}N_2O_9$
Exact Mass: 515.2969

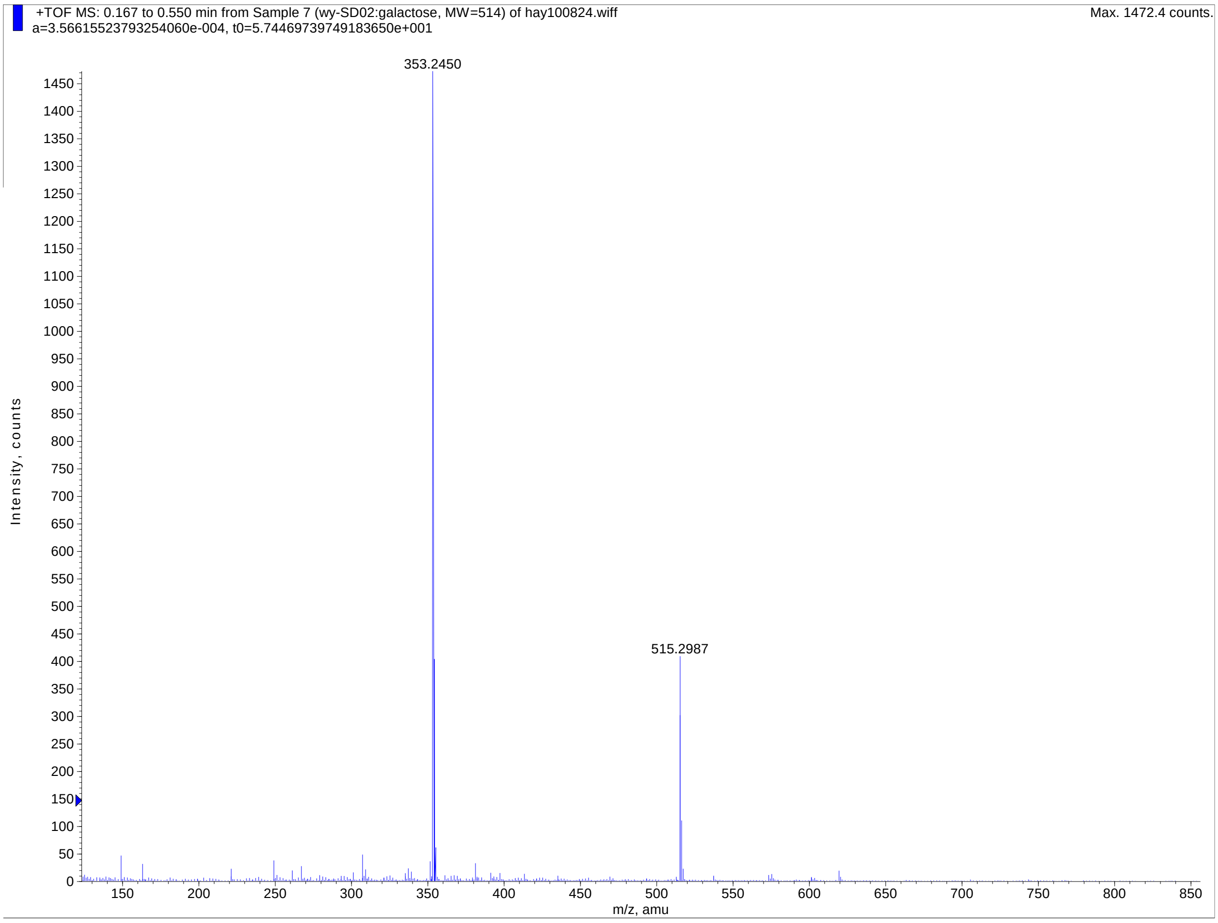

HRMS (ESI): calcd. For $C_{25}H_{42}N_2O_9H$ 515.2969 $[M+H]^+$, found 515.2987

iii. D-Mannose

Chemical Formula: $C_{25}H_{42}N_2O_9$
Exact Mass: 514.2890

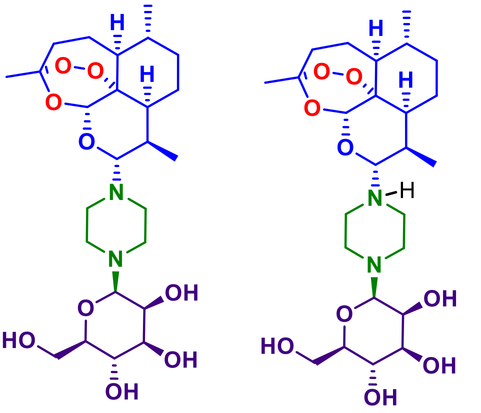

20

Chemical Formula: $C_{25}H_{43}N_2O_9$
Exact Mass: 515.2969

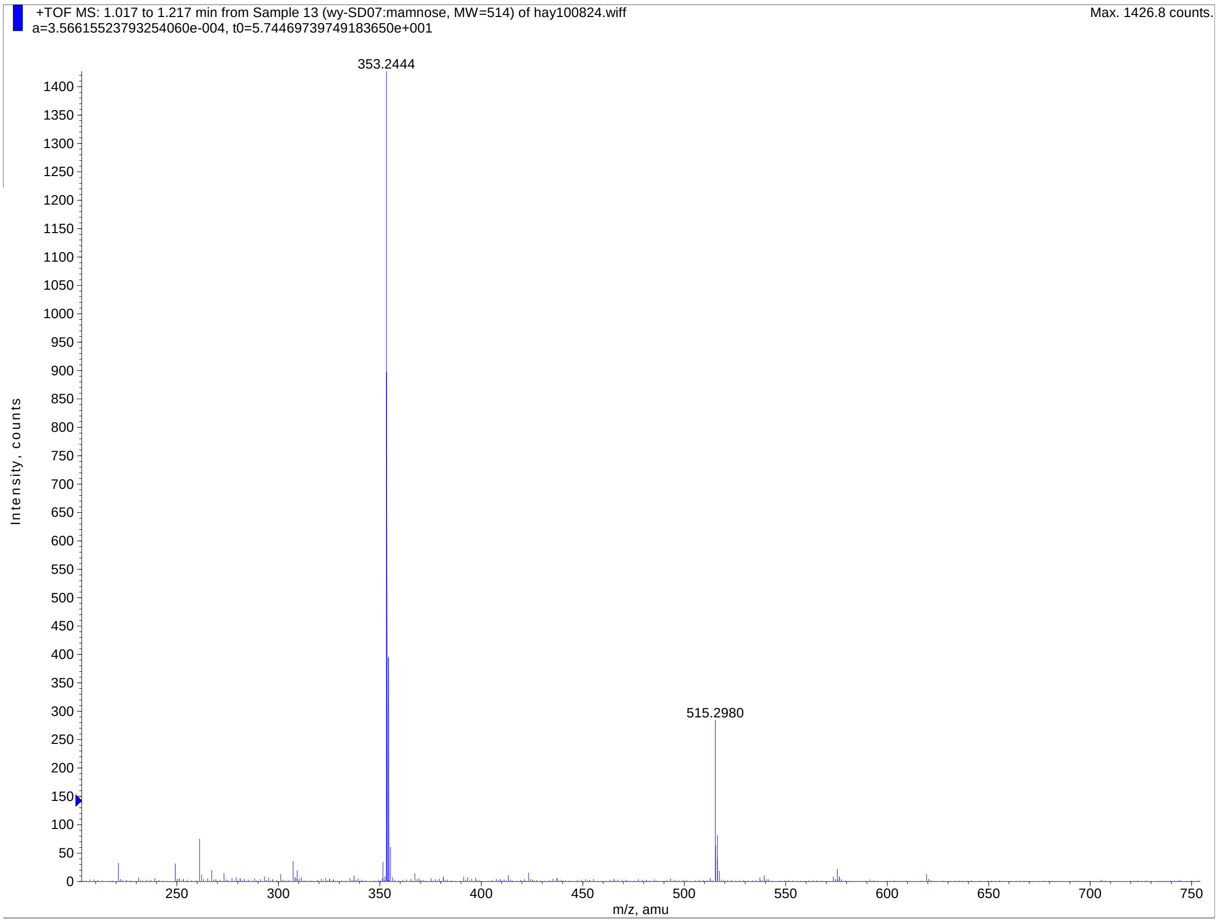

HRMS (ESI):): calcd. for $C_{25}H_{42}N_2O_9H$ 515.2969 $[M+H]^+$, found 515.2980.

iv. D-Maltose

Chemical Formula: $C_{31}H_{53}N_2O_{14}$
Exact Mass: 677.3497

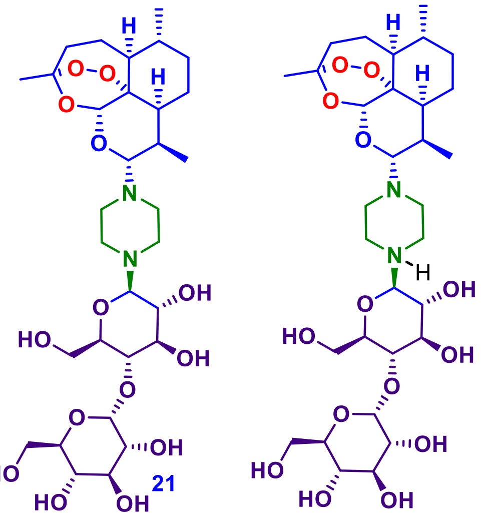

21

Chemical Formula: $C_{31}H_{52}N_2O_{14}$
Exact Mass: 676.3419

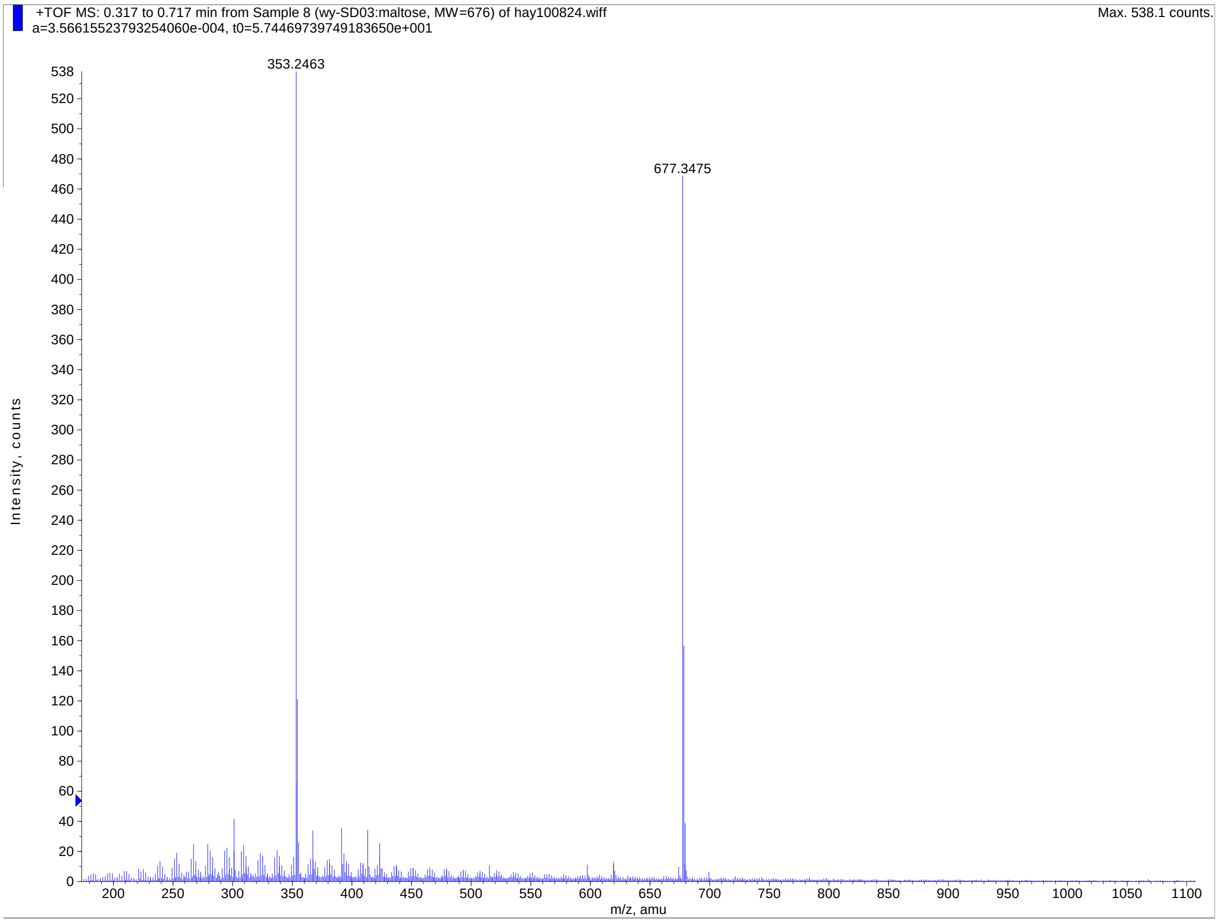

HRMS (ESI) calcd. for $C_{31}H_{53}N_2O_{14}$ 677.3497 $[M+H]^+$, found 677.3475.

v. 2-Deoxy-D-glucose

Chemical Formula: $C_{25}H_{42}N_2O_8$
Exact Mass: 498.2941

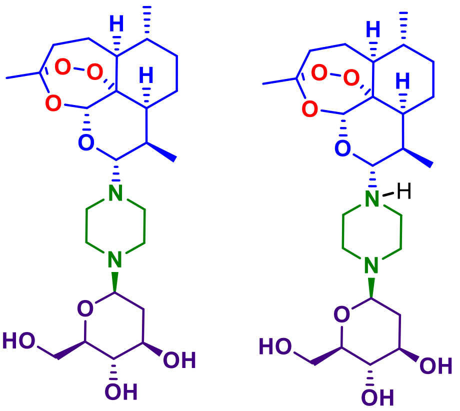

22

Chemical Formula: $C_{25}H_{43}N_2O_8$
Exact Mass: 499.3019

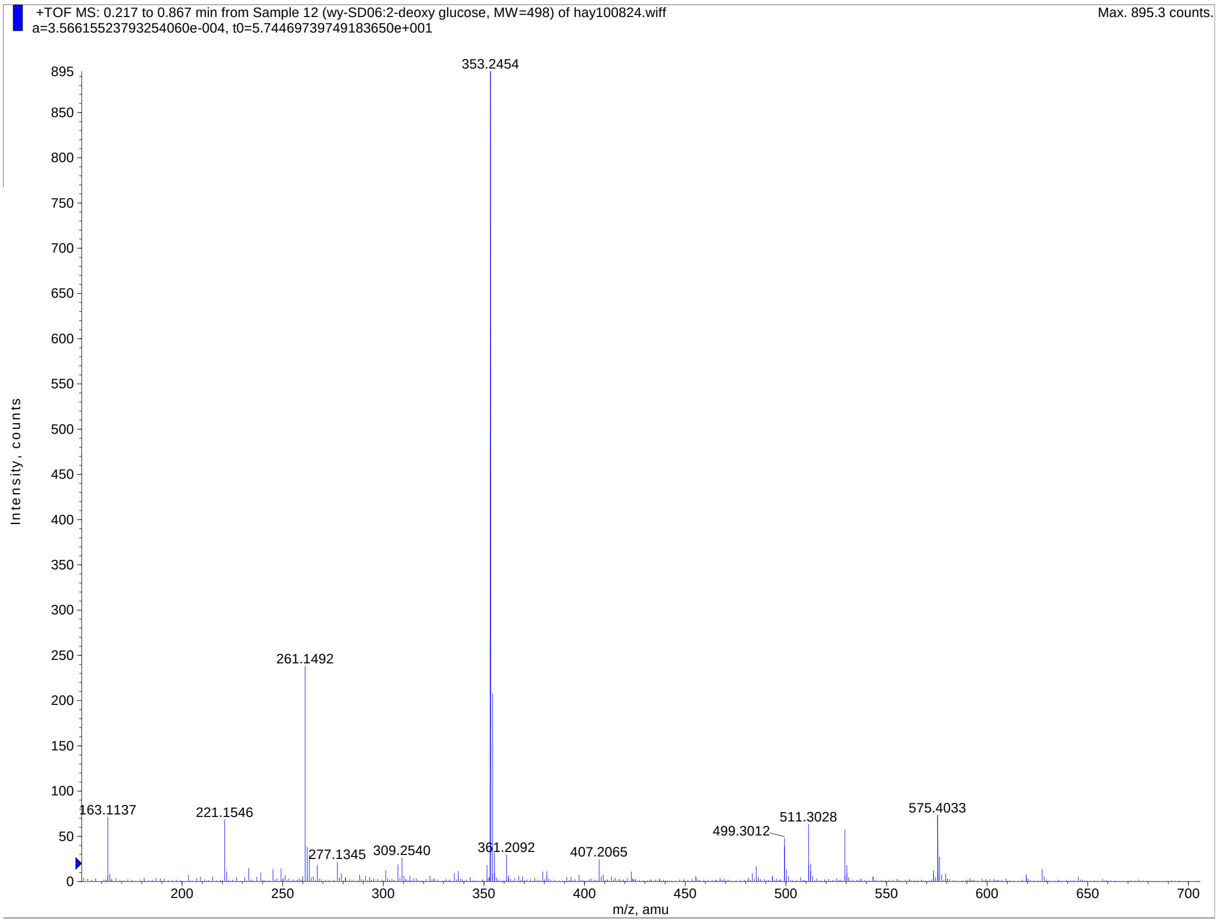

HRMS (ESI) calcd. for $C_{25}H_{43}N_2O_8$ 499.3019 $[M+H]^+$, found 499.3012

vi. L-Rhamnose

Chemical Formula: $C_{25}H_{42}N_2O_8$
Exact Mass: 498.2941

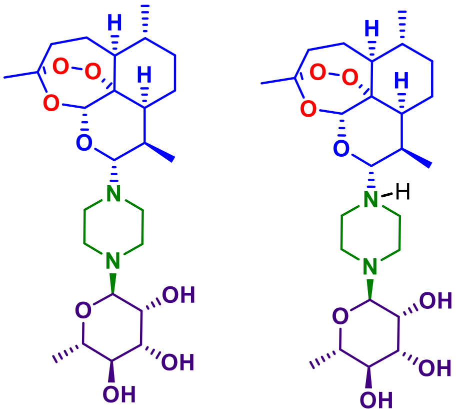

23

Chemical Formula: $C_{25}H_{43}N_2O_8$
Exact Mass: 499.3019

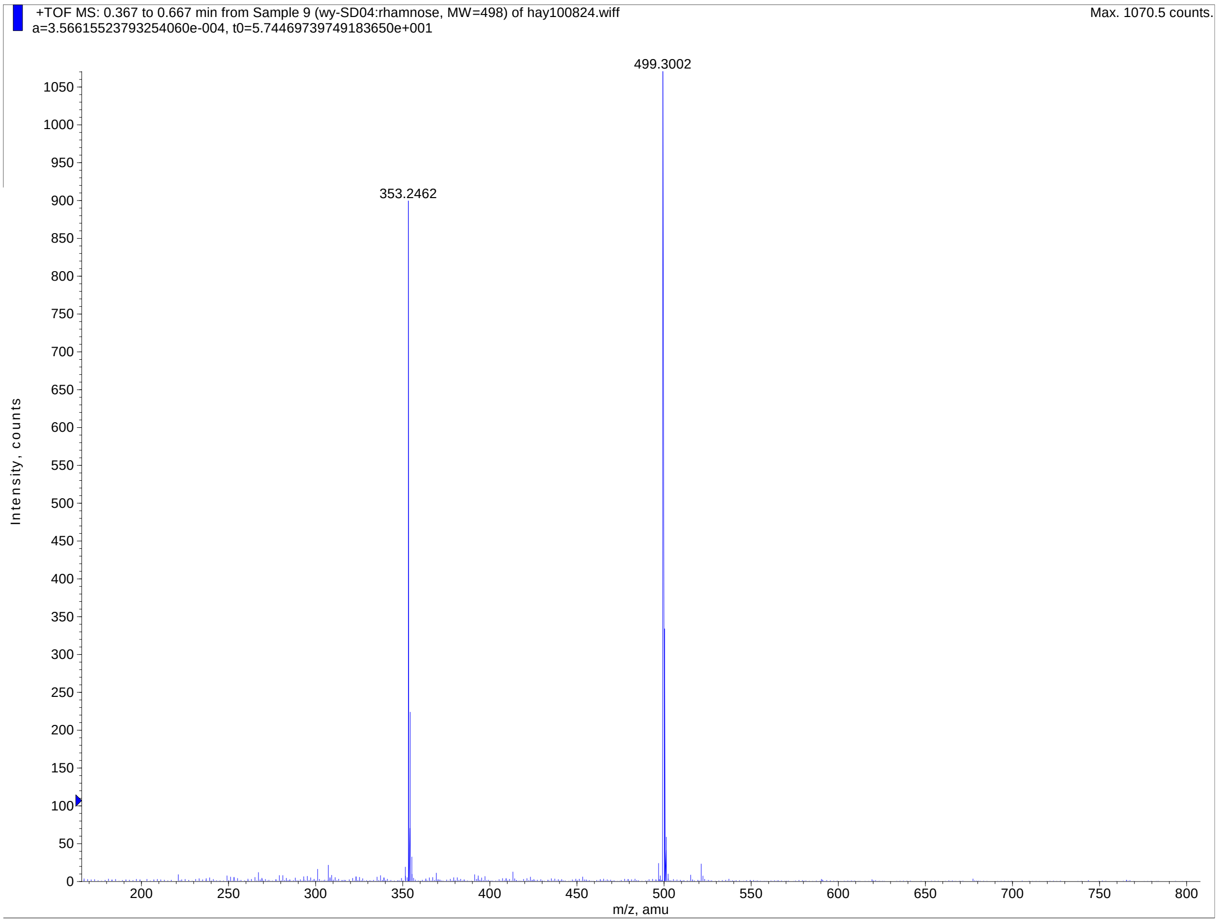

HRMS (ESI), calcd for $C_{25}H_{42}N_2O_8H^+$ 499.3019, m/z (M^++1) found 499.3002

vii. D-Ribose

Chemical Formula: $C_{24}H_{40}N_2O_8$
Exact Mass: 484.2785

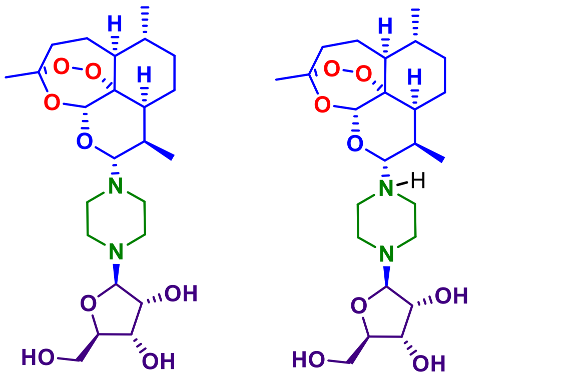

24 Chemical Formula: $C_{24}H_{41}N_2O_8$
Exact Mass: 485.2863

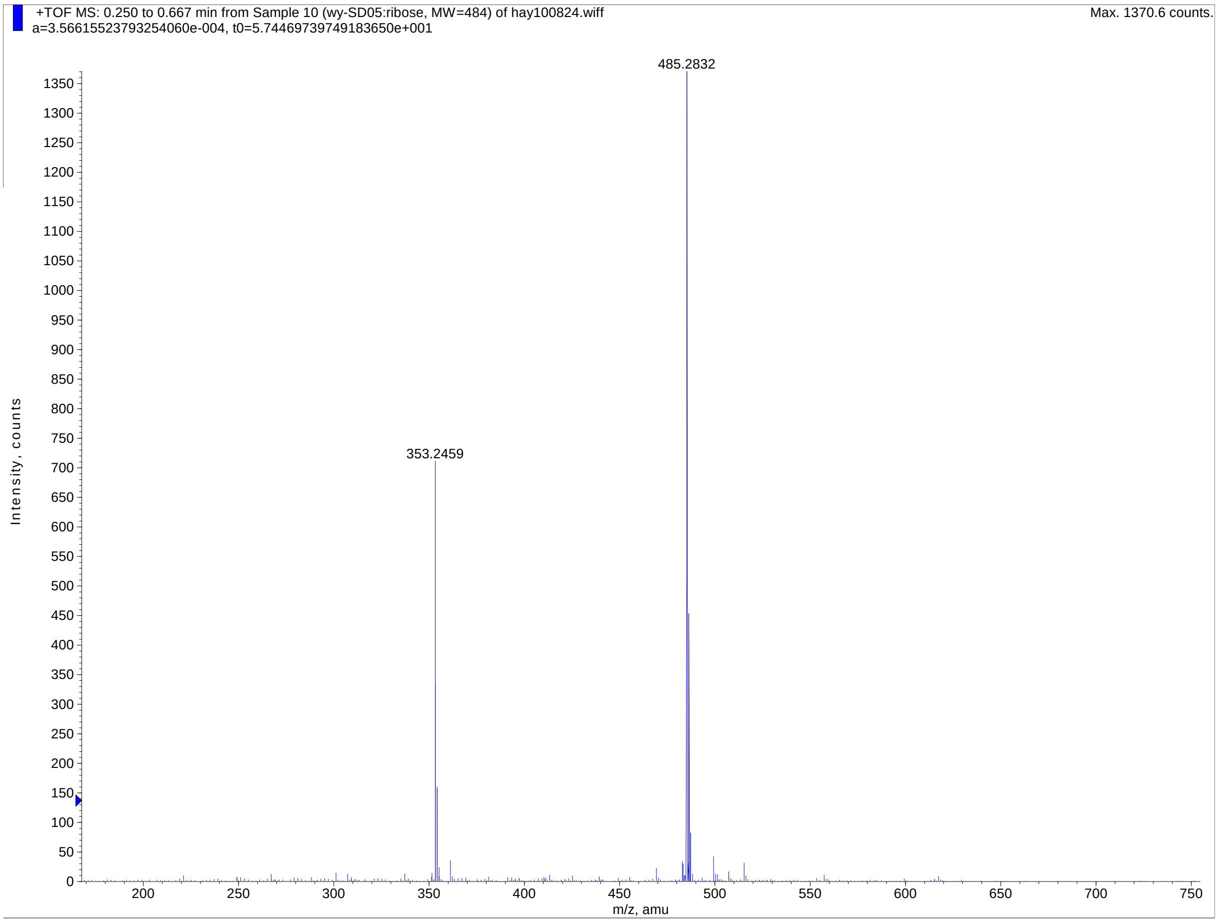

HRMS (ESI): calcd. for $C_{24}H_{41}N_2O_8$ 485.2863 $[M+H]^+$, found 485.2832.

viii. D-Xylose

Chemical Formula: $C_{24}H_{40}N_2O_8$
Exact Mass: 484.2785

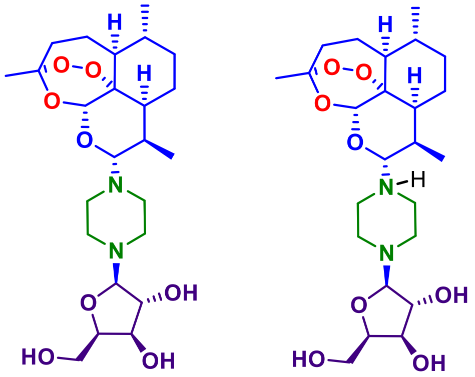

24 Chemical Formula: $C_{24}H_{41}N_2O_8$
Exact Mass: 485.2863

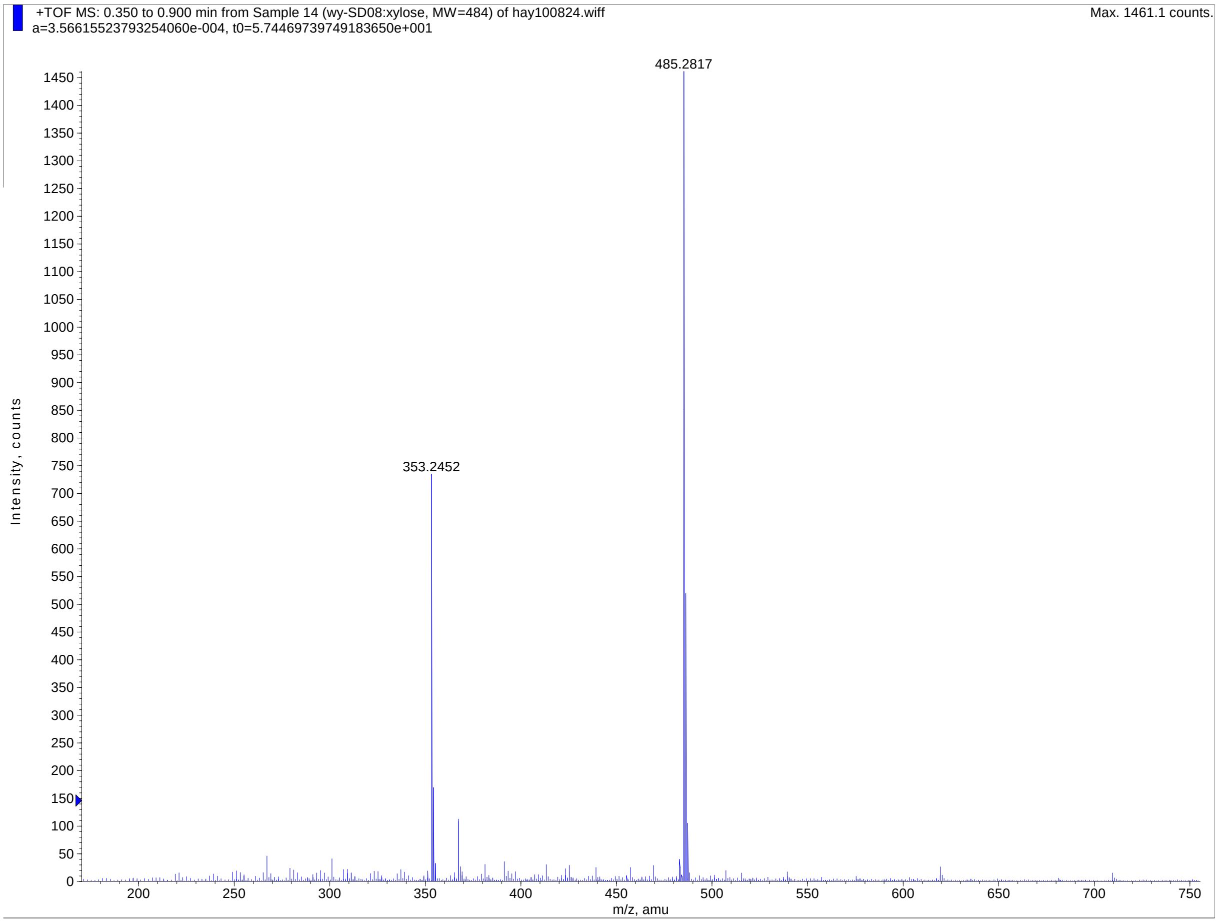

HRMS (ESI): calcd. for $C_{24}H_{41}N_2O_8$ 485.2863 $[M+H]^+$, found 485.2817.

ix. D-Arabinose

Chemical Formula: $C_{24}H_{40}N_2O_8$
Exact Mass: 484.2785

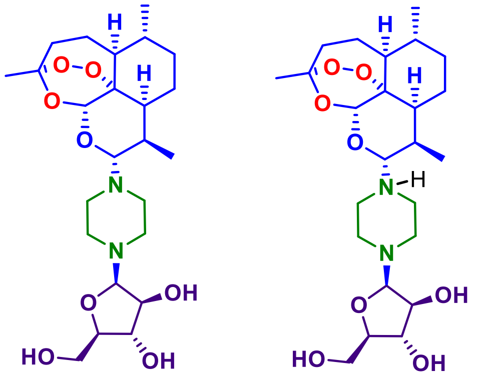

26 Chemical Formula: $C_{24}H_{41}N_2O_8$
Exact Mass: 485.2863

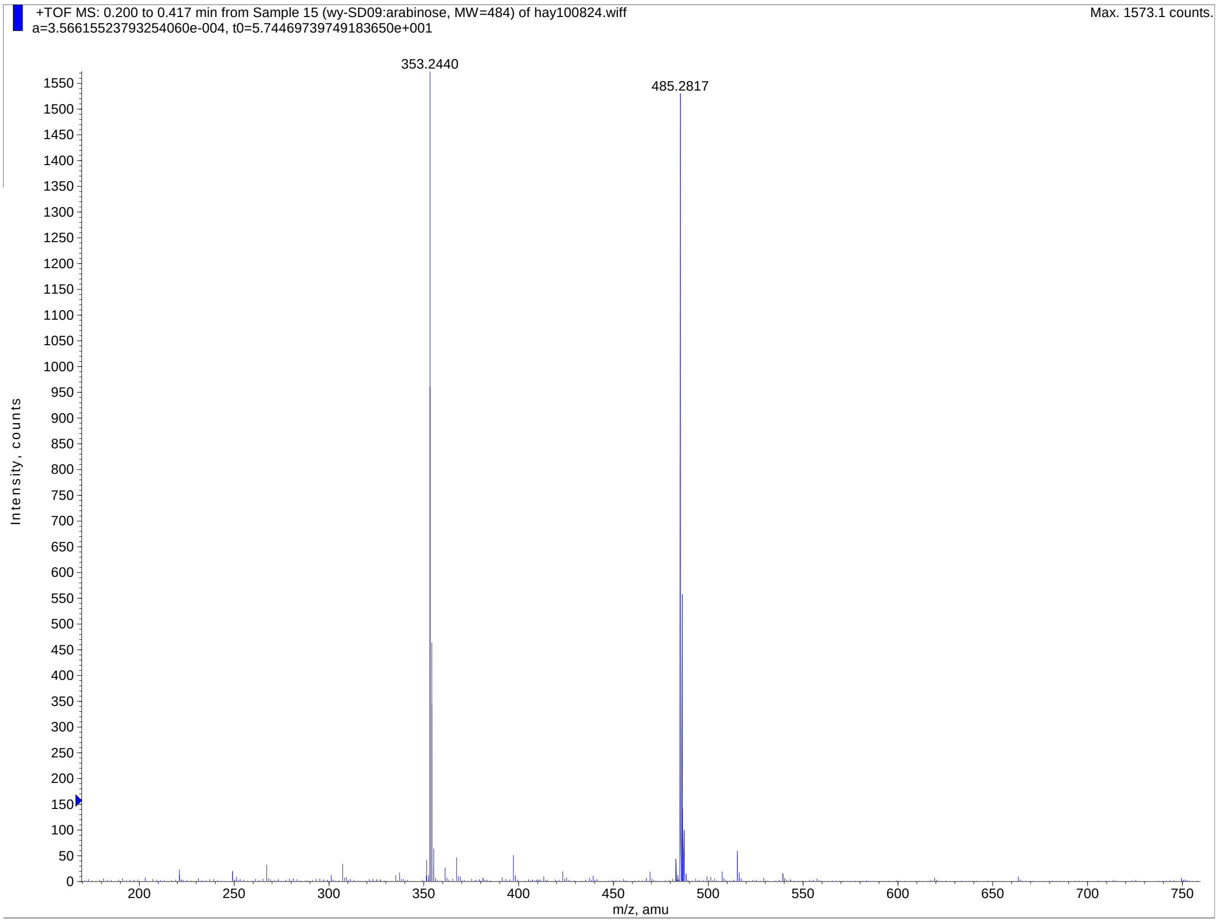

HRMS (ESI): calcd. for $C_{24}H_{41}N_2O_8$ 485.2863 $[M+H]^+$, found 485.2817

Crystallographic tables for compound **16** (Mich21)

Table S1. Crystal data and structure refinement for **16** (mich21)**.**

Identification code	mich21
Empirical formula	$C_{34}H_{54}N_2O_8$
Formula weight	618.82
Temperature/K	100.15
Crystal system	monoclinic
Space group	$P2_1$
a/Å	10.4611(12)
b/Å	14.6938(17)
c/Å	11.3606(12)
α/°	90
β/°	111.106(2)
γ/°	90
Volume/Å^3	1629.1(3)
Z	2
ρ_{calc}g/cm^3	1.2614
μ/mm^{-1}	0.089
F(000)	672.4
Crystal size/mm^3	0.18 × 0.06 × 0.04
Radiation	Mo Kα (λ = 0.71073)
2Θ range for data collection/°	4.18 to 50
Index ranges	$-13 \leq h \leq 12$, $-9 \leq k \leq 18$, $-14 \leq l \leq 13$
Reflections collected	9792
Independent reflections	2950 [R_{int} = 0.1049, R_{sigma} = 0.1577]
Data/restraints/parameters	2950/1/397
Goodness-of-fit on F^2	0.974
Final R indexes [I>=2σ (I)]	R_1 = 0.0499, wR_2 = 0.0601
Final R indexes [all data]	R_1= 0.0764, wR_2 = 0.1219
Largest diff. peak/hole / e Å^{-3}	0.59/-0.53

Table S2. Fractional Atomic Coordinates (×10^4) and Equivalent Isotropic Displacement Parameters (Å^2×10^3) for **16** (mich21). U_{eq} is defined as 1/3 of of the trace of the orthogonalised U_{IJ} tensor**.**

**Atom**	***x***	***y***	***z***	**U(eq)**
N1	4774(4)	1680(3)	3371(3)	18.1(11)
N2	3561(4)	2465(3)	888(3)	22.4(11)
C1	5614(5)	2096(4)	2710(4)	27.4(15)
C2	4874(5)	1983(4)	1312(4)	24.8(15)
C3	3484(5)	2188(4)	3009(4)	24.8(15)
C4	2714(5)	2114(4)	1587(4)	24.5(15)
O1A	8551(3)	2375(2)	6843(3)	22.0(9)
O2A	8101(3)	3326(2)	6471(3)	24.9(9)
O3A	6242(3)	3252(3)	7126(3)	24.9(10)
O4A	5643(3)	2425(2)	5340(3)	21.6(9)
O1B	3749(3)	1098(2)	-2552(3)	22.9(10)
O2B	3739(3)	304(2)	-1766(3)	23.5(9)
O3B	1399(3)	371(2)	-2146(3)	22.2(9)
O4B	2416(3)	1485(2)	-774(3)	21.5(10)
C1A	7545(5)	1863(4)	7176(4)	20.6(14)
C2A	6167(5)	2352(4)	6673(4)	24.3(14)
C3A	7541(5)	3668(4)	7358(5)	25.9(15)
C4A	8507(5)	3512(4)	8711(4)	27.9(15)
C5A	8164(5)	2669(4)	9304(5)	26.6(15)
C6A	7475(5)	1011(4)	9142(4)	31.0(16)
C7A	7481(6)	122(4)	8469(5)	35.4(17)
C8A	6743(5)	212(4)	7040(5)	27.5(15)
C9A	6803(5)	1065(4)	5064(4)	25.3(15)
C10A	5429(5)	1548(4)	4706(5)	25.8(15)
C11A	8140(5)	1768(4)	8631(4)	22.6(15)
C12A	7448(5)	937(4)	6506(4)	20.3(14)
C13A	7277(5)	4656(4)	7008(5)	31.8(15)
C14A	8227(6)	905(4)	10563(4)	52(2)
C15A	6688(5)	146(4)	4374(4)	31.3(16)
C1B	2457(5)	1610(4)	-2910(4)	20.2(15)
C2B	1677(5)	1310(4)	-2074(4)	22.5(15)

C3B	2471(5)	-160(4)	-2327(5)	25.1(15)
C4B	2169(5)	-305(4)	-3736(4)	29.1(15)
C5B	1197(5)	402(4)	-4561(5)	30.0(16)
C6B	509(5)	2062(4)	-4947(4)	30.7(16)
C7B	1009(5)	3033(4)	-4681(4)	30.3(16)
C8B	1635(5)	3228(4)	-3278(4)	26.9(15)
C9B	3635(5)	2761(4)	-1241(4)	21.4(14)
C10B	2813(5)	2422(4)	-466(4)	22.0(13)
C11B	1687(5)	1387(4)	-4307(4)	20.7(14)
C12B	2860(5)	2606(4)	-2667(4)	18.0(13)
C13B	2591(5)	-1016(4)	-1577(5)	32.8(17)
C14B	-183(5)	1892(4)	-6352(4)	32.4(16)
C15B	4036(5)	3756(4)	-952(5)	29.0(15)

Table S3. Anisotropic Displacement Parameters (Å^2×10^3) for **16** (mich21). The Anisotropic displacement factor exponent takes the form: $-2\pi^2[h^2a^{*2}U_{11}+2hka^*b^*U_{12}+\ldots]$.

**Atom**	**U_{11}**	**U_{22}**	**U_{33}**	**U_{12}**	**U_{13}**	**U_{23}**
N1	17(2)	23(3)	18(2)	3(2)	10(2)	-1(2)
N2	16(2)	28(3)	22(3)	10(2)	5(2)	6(2)
C1	20(3)	39(4)	24(3)	-2(3)	8(3)	1(3)
C2	22(3)	38(4)	13(3)	-7(3)	5(2)	-2(3)
C3	24(3)	33(4)	18(3)	-1(3)	9(3)	-3(3)
C4	19(3)	36(4)	16(3)	-2(3)	2(2)	-5(3)
O1A	18.4(19)	28(3)	21.4(19)	10(2)	8.9(16)	2(2)
O2A	28(2)	24(3)	26(2)	7(2)	13.6(17)	3(2)
O3A	20(2)	29(3)	26(2)	1(2)	9.2(17)	-6(2)
O4A	25(2)	24(3)	14.4(19)	-1(2)	6.2(16)	-5.0(19)
O1B	24(2)	24(3)	21(2)	3(2)	7.6(17)	6.3(19)
O2B	23(2)	22(3)	25(2)	2(2)	7.5(17)	8(2)
O3B	23(2)	23(3)	21(2)	-4(2)	7.7(17)	-5(2)
O4B	21(2)	26(3)	17(2)	-10(2)	6.6(17)	-2.4(18)
C1A	18(3)	25(4)	19(3)	-1(3)	7(3)	1(3)
C2A	29(3)	22(4)	23(3)	-5(3)	10(3)	2(3)
C3A	23(3)	33(4)	25(3)	-3(3)	12(3)	-8(3)

C4A	24(3)	33(4)	28(3)	1(3)	11(3)	-8(3)
C5A	31(3)	31(4)	21(3)	-8(3)	13(3)	-2(3)
C6A	34(4)	36(4)	24(3)	-4(4)	11(3)	-1(4)
C7A	42(4)	36(5)	29(3)	-7(4)	14(3)	7(3)
C8A	29(3)	21(4)	31(3)	-1(3)	9(3)	-2(3)
C9A	26(3)	28(4)	23(3)	-1(3)	9(3)	-5(3)
C10A	26(3)	30(4)	20(3)	-7(3)	7(3)	-8(3)
C11A	21(3)	25(4)	21(3)	-3(3)	7(3)	-8(3)
C12A	16(3)	23(4)	22(3)	-3(3)	7(2)	1(3)
C13A	26(3)	28(4)	42(4)	0(3)	12(3)	-7(3)
C14A	68(5)	60(5)	20(3)	-34(4)	7(3)	-7(4)
C15A	29(3)	36(4)	24(3)	6(3)	3(3)	-4(3)
C1B	11(3)	34(4)	17(3)	12(3)	7(2)	8(3)
C2B	23(3)	29(4)	17(3)	2(3)	9(3)	2(3)
C3B	22(3)	32(4)	21(3)	-5(3)	8(3)	-5(3)
C4B	31(3)	32(4)	25(3)	-5(3)	11(3)	-9(3)
C5B	22(3)	46(5)	23(3)	-1(3)	9(3)	-8(4)
C6B	29(3)	50(5)	12(3)	7(3)	6(3)	11(3)
C7B	24(3)	42(5)	22(3)	10(3)	4(3)	7(3)
C8B	17(3)	32(4)	29(3)	-4(3)	6(3)	3(3)
C9B	24(3)	17(4)	24(3)	-3(3)	10(3)	4(3)
C10B	23(3)	20(4)	18(3)	1(3)	1(2)	-1(3)
C11B	15(3)	33(4)	14(3)	-2(3)	5(2)	1(3)
C12B	14(3)	20(4)	19(3)	1(3)	4(2)	0(3)
C13B	41(4)	27(4)	35(4)	2(3)	19(3)	-2(3)
C14B	22(3)	55(5)	22(3)	3(3)	10(3)	4(3)
C15B	27(3)	31(4)	26(3)	-1(3)	6(3)	1(3)

Table S4. Bond Lengths for **16** (mich21).

**Atom**	**Atom**	**Length/Å**	**Atom**	**Atom**	**Length/Å**
N1	C1	1.478(5)	C3A	C13A	1.504(7)
N1	C3	1.465(5)	C4A	C5A	1.514(7)
N1	C10A	1.435(6)	C5A	C11A	1.525(7)
N2	C2	1.464(5)	C6A	C7A	1.515(7)

N2	C4	1.480(5)	C6A	C11A	1.533(7)
N2	C10B	1.455(5)	C6A	C14A	1.528(6)
C1	C2	1.505(6)	C7A	C8A	1.532(6)
C3	C4	1.528(6)	C8A	C12A	1.539(6)
O1A	O2A	1.486(5)	C9A	C10A	1.522(6)
O1A	C1A	1.452(5)	C9A	C12A	1.543(6)
O2A	C3A	1.427(5)	C9A	C15A	1.543(7)
O3A	C2A	1.411(6)	C1B	C2B	1.523(6)
O3A	C3A	1.426(6)	C1B	C11B	1.534(6)
O4A	C2A	1.417(5)	C1B	C12B	1.520(7)
O4A	C10A	1.453(6)	C3B	C4B	1.530(6)
O1B	O2B	1.472(4)	C3B	C13B	1.499(7)
O1B	C1B	1.470(5)	C4B	C5B	1.518(7)
O2B	C3B	1.422(5)	C5B	C11B	1.528(7)
O3B	C2B	1.406(6)	C6B	C7B	1.511(7)
O3B	C3B	1.441(5)	C6B	C11B	1.544(7)
O4B	C2B	1.422(5)	C6B	C14B	1.518(6)
O4B	C10B	1.443(6)	C7B	C8B	1.517(6)
C1A	C2A	1.525(6)	C8B	C12B	1.523(6)
C1A	C11A	1.548(6)	C9B	C10B	1.520(6)
C1A	C12A	1.544(7)	C9B	C12B	1.545(6)
C3A	C4A	1.523(7)	C9B	C15B	1.524(7)

Table S5. Bond Angles for **16** (mich21).

**Atom**	**Atom**	**Atom**	**Angle/°**	**Atom**	**Atom**	**Atom**	**Angle/°**
C3	N1	C1	107.6(4)	O4A	C10A	N1	109.5(4)
C10A	N1	C1	116.7(4)	C9A	C10A	N1	113.0(4)
C10A	N1	C3	114.0(4)	C9A	C10A	O4A	108.6(4)
C4	N2	C2	109.1(4)	C5A	C11A	C1A	112.6(5)
C10B	N2	C2	114.2(4)	C6A	C11A	C1A	114.0(4)
C10B	N2	C4	110.8(4)	C6A	C11A	C5A	111.6(4)
C2	C1	N1	108.5(4)	C8A	C12A	C1A	111.8(4)
C1	C2	N2	111.0(4)	C9A	C12A	C1A	110.1(4)
C4	C3	N1	110.0(4)	C9A	C12A	C8A	114.5(4)

C3	C4	N2	110.6(4)	C2B	C1B	O1B	109.0(4)
C1A	O1A	O2A	112.1(3)	C11B	C1B	O1B	105.4(4)
C3A	O2A	O1A	107.4(3)	C11B	C1B	C2B	112.5(4)
C3A	O3A	C2A	113.1(4)	C12B	C1B	O1B	106.0(4)
C10A	O4A	C2A	113.2(4)	C12B	C1B	C2B	109.9(4)
C1B	O1B	O2B	111.5(3)	C12B	C1B	C11B	113.7(4)
C3B	O2B	O1B	108.6(3)	O4B	C2B	O3B	105.2(4)
C3B	O3B	C2B	112.6(4)	C1B	C2B	O3B	113.2(4)
C10B	O4B	C2B	115.2(4)	C1B	C2B	O4B	112.5(4)
C2A	C1A	O1A	109.5(4)	O3B	C3B	O2B	108.9(4)
C11A	C1A	O1A	105.9(4)	C4B	C3B	O2B	110.1(4)
C11A	C1A	C2A	113.9(4)	C4B	C3B	O3B	110.3(4)
C12A	C1A	O1A	104.7(4)	C13B	C3B	O2B	105.9(4)
C12A	C1A	C2A	110.2(4)	C13B	C3B	O3B	106.5(4)
C12A	C1A	C11A	112.1(4)	C13B	C3B	C4B	114.9(5)
O4A	C2A	O3A	105.6(4)	C5B	C4B	C3B	113.0(4)
C1A	C2A	O3A	112.6(4)	C11B	C5B	C4B	115.3(4)
C1A	C2A	O4A	112.7(4)	C11B	C6B	C7B	110.6(4)
O3A	C3A	O2A	108.9(4)	C14B	C6B	C7B	111.5(5)
C4A	C3A	O2A	111.6(4)	C14B	C6B	C11B	112.8(5)
C4A	C3A	O3A	111.4(4)	C8B	C7B	C6B	112.1(5)
C13A	C3A	O2A	103.7(4)	C12B	C8B	C7B	109.8(4)
C13A	C3A	O3A	107.0(4)	C12B	C9B	C10B	111.4(4)
C13A	C3A	C4A	113.8(5)	C15B	C9B	C10B	111.1(4)
C5A	C4A	C3A	113.2(5)	C15B	C9B	C12B	111.6(4)
C11A	C5A	C4A	116.8(4)	O4B	C10B	N2	107.3(4)
C11A	C6A	C7A	110.5(4)	C9B	C10B	N2	113.4(4)
C14A	C6A	C7A	110.6(5)	C9B	C10B	O4B	110.2(4)
C14A	C6A	C11A	110.1(4)	C5B	C11B	C1B	114.4(4)
C8A	C7A	C6A	111.6(5)	C6B	C11B	C1B	112.5(4)
C12A	C8A	C7A	110.3(4)	C6B	C11B	C5B	111.3(4)
C12A	C9A	C10A	110.5(4)	C8B	C12B	C1B	111.2(4)
C15A	C9A	C10A	112.3(4)	C9B	C12B	C1B	109.9(4)
C15A	C9A	C12A	110.9(5)	C9B	C12B	C8B	115.3(4)

Table S6. Hydrogen Atom Coordinates (Å×10^4) and Isotropic Displacement Parameters (Å^2×10^3) for **16** (mich21).

**Atom**	***x***	***y***	***z***	**U(eq)**
H1a	5759(5)	2750(4)	2925(4)	32.9(18)
H1b	6520(5)	1794(4)	2972(4)	32.9(18)
H2a	4712(5)	1328(4)	1109(4)	29.7(18)
H2b	5451(5)	2223(4)	857(4)	29.7(18)
H3a	2911(5)	1941(4)	3463(4)	29.8(18)
H3b	3677(5)	2836(4)	3248(4)	29.8(18)
H4a	1851(5)	2466(4)	1349(4)	29.4(18)
H4b	2476(5)	1469(4)	1358(4)	29.4(18)
H2Aa	5495(5)	2005(4)	6940(4)	29.1(17)
H4Aa	9456(5)	3458(4)	8724(4)	33.5(18)
H4Ab	8472(5)	4049(4)	9226(4)	33.5(18)
H5Aa	8839(5)	2616(4)	10175(5)	32.0(18)
H5Ab	7253(5)	2759(4)	9367(5)	32.0(18)
H6A	6503(5)	1185(4)	8985(4)	37.1(19)
H7Aa	8439(6)	-71(4)	8649(5)	42(2)
H7Ab	7025(6)	-354(4)	8795(5)	42(2)
H8Aa	6754(5)	-381(4)	6630(5)	33.0(17)
H8Ab	5775(5)	386(4)	6853(5)	33.0(17)
H9A	7429(5)	1464(4)	4803(4)	30.4(18)
H10A	4810(5)	1175(4)	5011(5)	30.9(18)
H11A	9120(5)	1586(4)	8852(4)	27.1(17)
H12A	8409(5)	725(4)	6695(4)	24.4(17)
H13a	8150(5)	4966(4)	7155(5)	48(2)
H13b	6697(5)	4706(4)	6115(5)	48(2)
H13c	6813(5)	4938(4)	7526(5)	48(2)
H14a	8213(6)	1486(4)	10983(4)	78(3)
H14b	7772(6)	437(4)	10887(4)	78(3)
H14c	9178(6)	724(4)	10731(4)	78(3)
H15a	7593(5)	-140(4)	4632(4)	47(2)
H15b	6057(5)	-254(4)	4592(4)	47(2)
H15c	6340(5)	249(4)	3461(4)	47(2)
H2Ba	789(5)	1648(4)	-2337(4)	27.1(18)

H4Ba	3041(5)	-286(4)	-3890(4)	34.9(19)
H4Bb	1763(5)	-917(4)	-3979(4)	34.9(19)
H5Ba	1037(5)	259(4)	-5454(5)	36.0(19)
H5Bb	307(5)	349(4)	-4444(5)	36.0(19)
H6B	-198(5)	1971(4)	-4556(4)	36.8(19)
H7Ba	1701(5)	3148(4)	-5070(4)	36(2)
H7Bb	231(5)	3452(4)	-5073(4)	36(2)
H8Ba	1931(5)	3871(4)	-3142(4)	32.2(18)
H8Bb	942(5)	3127(4)	-2884(4)	32.2(18)
H9B	4500(5)	2398(4)	-988(4)	25.7(17)
H10B	1965(5)	2800(4)	-675(4)	26.4(16)
H11B	2364(5)	1473(4)	-4734(4)	24.8(17)
H12B	3527(5)	2732(4)	-3094(4)	21.6(16)
H13d	1739(5)	-1366(4)	-1924(5)	49(2)
H13e	3356(5)	-1382(4)	-1621(5)	49(2)
H13f	2755(5)	-861(4)	-695(5)	49(2)
H14d	-495(5)	1258(4)	-6493(4)	49(2)
H14e	-971(5)	2300(4)	-6697(4)	49(2)
H14f	470(5)	2006(4)	-6774(4)	49(2)
H15d	4565(5)	3958(4)	-1462(5)	44(2)
H15e	3207(5)	4128(4)	-1152(5)	44(2)
H15f	4595(5)	3822(4)	-54(5)	44(2)

Experimental

Single crystals of compound **16** [**mich21**] $C_{34}H_{54}N_2O_8$ were grown from ethyl acetate-hexane. A suitable crystal was selected and mounted on a diffractometer. The crystal was kept at 100.15 K during data collection. Using Olex2 [1], the structure was solved with the ShelXS [2] structure solution program using Direct Methods and refined with the olex2.refine [3] refinement package using Gauss-Newton minimisation.

1. Dolomanov, O.V., Bourhis, L.J., Gildea, R.J, Howard, J.A.K. & Puschmann, H. (2009), J. Appl. Cryst. 42, 339-341.
2. Sheldrick, G.M. (2008). Acta Cryst. A64, 112-122.
3. Bourhis, L.J., Dolomanov, O.V., Gildea, R.J., Howard, J.A.K., Puschmann, H. (2015). Acta Cryst. A71, 59-75.

Crystal structure determination of compound 16 [mich21]

Crystal Data for $C_{34}H_{54}N_2O_8$ (M =618.82 g/mol): monoclinic, space group $P2_1$ (no. 4), a = 10.4611(12) Å, b = 14.6938(17) Å, c = 11.3606(12) Å, β = 111.106(2)°, V = 1629.1(3) Å^3, Z = 2, T = 100.15 K, μ(Mo Kα) = 0.089 mm^{-1}, *Dcalc* = 1.2614 g/cm^3, 9792 reflections measured (4.18° ≤ 2Θ ≤ 50°), 2950 unique (R_{int} = 0.1049, R_{sigma} = 0.1577) which were used in all calculations. The final R_1 was 0.0499 (I>=2u(I)) and wR_2 was 0.0764 (all data).

Refinement model description

Number of restraints - 1, number of constraints - 84.

Details:

```
1. Fixed Uiso
 At 1.2 times of:
  All C(H) groups, All C(H,H) groups
 At 1.5 times of:
  All C(H,H,H) groups
2.a Ternary CH refined with riding coordinates:
 C2A(H2Aa), C6A(H6A), C9A(H9A), C10A(H10A), C11A(H11A), C12A(H12A), C2B(H2Ba),
 C6B(H6B), C9B(H9B), C10B(H10B), C11B(H11B), C12B(H12B)
2.b Secondary CH2 refined with riding coordinates:
 C1(H1a,H1b), C2(H2a,H2b), C3(H3a,H3b), C4(H4a,H4b), C4A(H4Aa,H4Ab), C5A(H5Aa,
 H5Ab), C7A(H7Aa,H7Ab), C8A(H8Aa,H8Ab), C4B(H4Ba,H4Bb), C5B(H5Ba,H5Bb),
 C7B(H7Ba,H7Bb), C8B(H8Ba,H8Bb)
2.c Me refined with riding coordinates:
 C13A(H13a,H13b,H13c), C14A(H14a,H14b,H14c), C15A(H15a,H15b,H15c), C13B(H13d,
 H13e,H13f), C14B(H14d,H14e,H14f), C15B(H15d,H15e,H15f)
```

This report has been created with Olex2, compiled on 2017.08.10 svn.r3458 for OlexSys.

Crystallographic tables for compound 18 (yuet5)

Table S7. Crystal data and structure refinement for yuet5.

Identification code	yuet5	
Empirical formula	$C_{25}H_{44}N_2O_{10}$	
Formula weight	532.62	
Temperature	173(2) K	
Wavelength	1.54178 Å	
Crystal system	Monoclinic	
Space group	P2(1)	
Unit cell dimensions	a = 9.1468(3) Å	α = 90°.
	b = 6.2120(2) Å	ß = 94.628(3)°.
	c = 23.5247(7) Å	γ = 90°.
Volume	1332.31(7) $Å^3$	
Z	2	
Density (calculated)	1.328 Mg/m^3	
Absorption coefficient	0.850 mm^{-1}	
F(000)	576	
Crystal size	0.40 x 0.03 x 0.02 mm^3	
Theta range for data collection	3.77 to 67.48°.	
Index ranges	-10<=h<=10, -7<=k<=7, -28<=l<=28	
Reflections collected	16629	
Independent reflections	4723 [R(int) = 0.0552]	
Completeness to theta = 66.50°	98.4 %	
Absorption correction	Semi-empirical from equivalents	
Max. and min. transmission	1.00 and 0.70	
Refinement method	Full-matrix least-squares on F^2	
Data / restraints / parameters	4723 / 1 / 337	
Goodness-of-fit on F^2	1.008	
Final R indices [I>2sigma(I)]	R1 = 0.0496, wR2 = 0.1255	
R indices (all data)	R1 = 0.0558, wR2 = 0.1290	
Absolute structure parameter	-0.01(19)	
Largest diff. peak and hole	0.337 and -0.258 e.$Å^{-3}$	

Table S8. Atomic coordinates (x 10^4) and equivalent isotropic displacement parameters ($Å^2$x 10^3) for **18** (yuet5). U(eq) is defined as one third of the trace of the orthogonalized U^{ij} tensor.

	x	y	z	U(eq)
O(1)	5058(3)	5607(4)	3496(1)	55(1)
O(2)	5328(3)	4886(3)	2916(1)	54(1)
O(3)	6767(2)	7796(3)	2701(1)	44(1)
O(4)	4509(2)	9144(3)	2688(1)	42(1)
O(20)	-2131(2)	9456(2)	1246(1)	27(1)
O(22)	444(2)	6618(3)	391(1)	29(1)
O(23)	-2433(2)	4837(3)	14(1)	31(1)
O(24)	-4498(2)	8277(3)	-51(1)	29(1)
O(26)	-5038(2)	8388(3)	1471(1)	36(1)
N(1)	2025(2)	10058(3)	2505(1)	34(1)
N(2)	441(2)	9245(3)	1422(1)	27(1)
C(1)	5582(4)	7797(5)	3605(1)	47(1)
C(2)	5820(3)	8913(4)	3040(1)	40(1)
C(3)	6753(4)	5489(5)	2787(1)	49(1)
C(4)	7930(5)	4882(6)	3260(2)	63(1)
C(5)	8280(4)	6640(6)	3697(2)	62(1)
C(6)	7440(4)	9641(6)	4334(1)	58(1)
C(7)	6132(5)	10697(6)	4597(1)	61(1)
C(8)	4847(4)	11043(5)	4174(1)	55(1)
C(9)	2935(4)	9089(6)	3486(1)	55(1)
C(10)	3240(3)	10169(5)	2926(1)	39(1)
C(11)	6945(4)	4449(6)	2219(2)	60(1)
C(12)	8668(5)	9198(8)	4794(2)	74(1)
C(13)	1699(5)	10346(11)	3747(2)	85(1)
C(14)	1704(3)	7854(4)	2298(1)	35(1)
C(15)	304(3)	7840(4)	1913(1)	33(1)
C(16)	2140(3)	11506(4)	2011(1)	33(1)
C(17)	742(3)	11454(4)	1626(1)	30(1)
C(21)	-762(2)	9136(3)	992(1)	24(1)
C(22)	-837(3)	6971(3)	680(1)	25(1)
C(23)	-2117(3)	6964(3)	225(1)	25(1)
C(24)	-3535(2)	7788(3)	441(1)	24(1)
C(25)	-3319(3)	9765(3)	825(1)	26(1)

C(26)	-4667(3)	10206(4)	1140(1)	31(1)
C(51)	7000(4)	7561(5)	4007(1)	56(1)
C(81)	4327(4)	8905(5)	3899(1)	53(1)
O(1W)	3012(2)	5328(3)	973(1)	39(1)

Table S9. Bond lengths [Å] and angles [°] for **18** (yuet5).

O(1)-C(1)	1.458(4)
O(1)-O(2)	1.476(3)
O(2)-C(3)	1.413(4)
O(3)-C(2)	1.408(3)
O(3)-C(3)	1.448(4)
O(4)-C(2)	1.409(3)
O(4)-C(10)	1.474(4)
O(20)-C(25)	1.424(3)
O(20)-C(21)	1.444(3)
O(22)-C(22)	1.418(3)
O(23)-C(23)	1.433(3)
O(24)-C(24)	1.429(3)
O(26)-C(26)	1.428(3)
N(1)-C(10)	1.429(3)
N(1)-C(14)	1.475(3)
N(1)-C(16)	1.479(3)
N(2)-C(21)	1.435(3)
N(2)-C(15)	1.461(3)
N(2)-C(17)	1.472(3)
C(1)-C(2)	1.529(4)
C(1)-C(81)	1.549(5)
C(1)-C(51)	1.549(5)
C(3)-C(11)	1.506(5)
C(3)-C(4)	1.532(5)
C(4)-C(5)	1.516(5)
C(5)-C(51)	1.539(6)
C(6)-C(12)	1.521(5)
C(6)-C(7)	1.538(6)
C(6)-C(51)	1.540(5)
C(7)-C(8)	1.493(5)

C(8)-C(81)	1.536(4)
C(9)-C(10)	1.526(4)
C(9)-C(13)	1.541(6)
C(9)-C(81)	1.542(5)
C(14)-C(15)	1.508(3)
C(16)-C(17)	1.507(3)
C(21)-C(22)	1.531(3)
C(22)-C(23)	1.520(3)
C(23)-C(24)	1.519(3)
C(24)-C(25)	1.527(3)
C(25)-C(26)	1.514(3)
C(1)-O(1)-O(2)	111.5(2)
C(3)-O(2)-O(1)	110.2(2)
C(2)-O(3)-C(3)	113.4(2)
C(2)-O(4)-C(10)	118.63(19)
C(25)-O(20)-C(21)	111.63(16)
C(10)-N(1)-C(14)	113.3(2)
C(10)-N(1)-C(16)	114.5(2)
C(14)-N(1)-C(16)	109.36(18)
C(21)-N(2)-C(15)	114.75(18)
C(21)-N(2)-C(17)	112.69(18)
C(15)-N(2)-C(17)	109.00(18)
O(1)-C(1)-C(2)	109.8(2)
O(1)-C(1)-C(81)	104.3(3)
C(2)-C(1)-C(81)	110.4(2)
O(1)-C(1)-C(51)	105.4(2)
C(2)-C(1)-C(51)	113.8(3)
C(81)-C(1)-C(51)	112.5(3)
O(3)-C(2)-O(4)	104.5(2)
O(3)-C(2)-C(1)	113.9(2)
O(4)-C(2)-C(1)	112.6(2)
O(2)-C(3)-O(3)	108.1(2)
O(2)-C(3)-C(11)	104.6(3)
O(3)-C(3)-C(11)	107.4(3)
O(2)-C(3)-C(4)	112.6(3)
O(3)-C(3)-C(4)	109.4(2)
C(11)-C(3)-C(4)	114.4(3)

C(5)-C(4)-C(3)	114.4(3)
C(4)-C(5)-C(51)	117.5(3)
C(12)-C(6)-C(7)	110.2(3)
C(12)-C(6)-C(51)	110.6(3)
C(7)-C(6)-C(51)	112.2(3)
C(8)-C(7)-C(6)	112.9(3)
C(7)-C(8)-C(81)	110.9(3)
C(10)-C(9)-C(13)	108.1(3)
C(10)-C(9)-C(81)	112.2(3)
C(13)-C(9)-C(81)	112.5(3)
N(1)-C(10)-O(4)	108.0(2)
N(1)-C(10)-C(9)	113.1(2)
O(4)-C(10)-C(9)	110.1(2)
N(1)-C(14)-C(15)	109.9(2)
N(2)-C(15)-C(14)	110.4(2)
N(1)-C(16)-C(17)	110.38(19)
N(2)-C(17)-C(16)	110.06(19)
N(2)-C(21)-O(20)	110.06(17)
N(2)-C(21)-C(22)	112.46(18)
O(20)-C(21)-C(22)	108.24(17)
O(22)-C(22)-C(23)	106.37(17)
O(22)-C(22)-C(21)	111.04(18)
C(23)-C(22)-C(21)	110.03(17)
O(23)-C(23)-C(24)	105.83(18)
O(23)-C(23)-C(22)	111.65(17)
C(24)-C(23)-C(22)	113.15(18)
O(24)-C(24)-C(23)	106.73(18)
O(24)-C(24)-C(25)	110.47(17)
C(23)-C(24)-C(25)	113.30(18)
O(20)-C(25)-C(26)	106.81(18)
O(20)-C(25)-C(24)	111.00(16)
C(26)-C(25)-C(24)	111.30(19)
O(26)-C(26)-C(25)	111.28(18)
C(5)-C(51)-C(6)	111.7(3)
C(5)-C(51)-C(1)	112.1(3)
C(6)-C(51)-C(1)	113.4(3)
C(8)-C(81)-C(9)	114.4(3)
C(8)-C(81)-C(1)	110.9(3)

C(9)-C(81)-C(1)		110.8(2)

Symmetry transformations used to generate equivalent atoms

Table S10. Anisotropic displacement parameters ($Å^2$ x 10^3)for **18** (yuet5). The anisotropic displacement factor exponent takes the form: $-2\pi^2[h^2a^{*2}U^{11} + ... + 2 h k a^* b^* U^{12}]$

	U^{11}	U^{22}	U^{33}	U^{23}	U^{13}	U^{12}
O(1)	73(2)	42(1)	49(1)	1(1)	-2(1)	-19(1)
O(2)	63(2)	42(1)	55(1)	-6(1)	1(1)	-12(1)
O(3)	42(1)	38(1)	51(1)	0(1)	-1(1)	-2(1)
O(4)	38(1)	46(1)	42(1)	5(1)	-6(1)	-4(1)
O(20)	21(1)	29(1)	30(1)	-4(1)	-5(1)	2(1)
O(22)	19(1)	32(1)	35(1)	-5(1)	-2(1)	4(1)
O(23)	24(1)	25(1)	42(1)	-12(1)	-2(1)	1(1)
O(24)	22(1)	31(1)	34(1)	-2(1)	-6(1)	4(1)
O(26)	35(1)	41(1)	31(1)	-3(1)	2(1)	0(1)
N(1)	34(1)	34(1)	32(1)	-3(1)	-6(1)	-8(1)
N(2)	24(1)	22(1)	34(1)	1(1)	-8(1)	-1(1)
C(1)	56(2)	39(1)	44(2)	1(1)	-9(1)	-12(1)
C(2)	39(2)	37(1)	42(1)	0(1)	-8(1)	-5(1)
C(3)	45(2)	35(1)	64(2)	-3(1)	-3(1)	-6(1)
C(4)	67(2)	45(2)	74(2)	2(2)	-9(2)	-2(2)
C(5)	59(2)	58(2)	64(2)	7(2)	-17(2)	1(2)
C(6)	77(3)	53(2)	42(2)	6(1)	-15(2)	-13(2)
C(7)	81(3)	61(2)	38(2)	-3(1)	-14(2)	-20(2)
C(8)	73(2)	55(2)	34(1)	-6(1)	-11(2)	-13(2)
C(9)	56(2)	73(2)	33(1)	0(1)	-1(1)	-21(2)
C(10)	38(2)	46(1)	32(1)	-3(1)	-6(1)	-10(1)
C(11)	61(2)	47(2)	71(2)	-11(2)	9(2)	3(2)
C(12)	82(3)	82(3)	53(2)	7(2)	-20(2)	-19(2)
C(13)	61(3)	141(4)	53(2)	-14(3)	11(2)	-17(3)
C(14)	35(1)	30(1)	37(1)	1(1)	-9(1)	0(1)
C(15)	36(1)	27(1)	34(1)	3(1)	-9(1)	-5(1)
C(16)	30(1)	29(1)	38(1)	-4(1)	-4(1)	-6(1)
C(17)	28(1)	22(1)	37(1)	-2(1)	-4(1)	-2(1)

C(21)	16(1)	23(1)	32(1)	1(1)	-3(1)	1(1)
C(22)	21(1)	22(1)	31(1)	0(1)	-4(1)	1(1)
C(23)	23(1)	20(1)	32(1)	-1(1)	-2(1)	-4(1)
C(24)	20(1)	23(1)	30(1)	-1(1)	-3(1)	0(1)
C(25)	24(1)	22(1)	31(1)	-2(1)	-4(1)	2(1)
C(26)	28(1)	30(1)	34(1)	-5(1)	-4(1)	6(1)
C(51)	64(2)	49(2)	50(2)	12(1)	-16(2)	-12(2)
C(81)	68(2)	56(2)	34(1)	4(1)	-8(1)	-18(2)
O(1W)	30(1)	38(1)	48(1)	-6(1)	-4(1)	1(1)

Table S11. Hydrogen coordinates (x 10^4) and isotropic displacement parameters (Å^{2}x 10^3) for **18** (yuet5).

	x	y	z	U(eq)
H(22D)	1186	6492	630	35
H(23D)	-1639	4314	-90	37
H(24D)	-5357	8548	43	35
H(26D)	-5147	8751	1813	43
H(2A)	6238	10377	3125	48
H(4A)	7599	3588	3459	75
H(4B)	8841	4497	3083	75
H(5A)	8744	7845	3503	74
H(5B)	9020	6069	3989	74
H(6A)	7818	10683	4056	70
H(7A)	5829	9773	4909	73
H(7B)	6446	12101	4764	73
H(8A)	5123	12048	3874	66
H(8B)	4035	11703	4367	66
H(9A)	2569	7599	3398	65
H(10A)	3478	11718	3003	47
H(11A)	6071	4712	1959	89
H(11B)	7083	2895	2272	89
H(11C)	7806	5063	2057	89
H(12A)	9529	8651	4617	111
H(12B)	8340	8123	5061	111
H(12C)	8926	10533	5000	111

H(13A)	766	10064	3525	127
H(13B)	1915	11891	3738	127
H(13C)	1631	9884	4142	127
H(14A)	1603	6884	2627	42
H(14B)	2524	7321	2087	42
H(15A)	86	6354	1780	39
H(15B)	-519	8337	2128	39
H(16A)	2972	11050	1795	39
H(16B)	2332	12995	2148	39
H(17A)	-84	11971	1837	36
H(17B)	834	12421	1296	36
H(21A)	-645	10306	708	28
H(22A)	-959	5780	958	30
H(23A)	-1860	7892	-100	30
H(24A)	-3981	6612	660	29
H(25A)	-3112	11045	586	31
H(26A)	-4481	11463	1394	37
H(26B)	-5502	10561	862	37
H(51A)	6779	6473	4301	67
H(81A)	4078	7935	4216	64
H(1W)	3539	6246	1163	47
H(2W)	3549	4649	755	47

Table S12. Torsion angles [°] for **18** (yuet5).

C(1)-O(1)-O(2)-C(3)	43.5(3)
O(2)-O(1)-C(1)-C(2)	17.2(3)
O(2)-O(1)-C(1)-C(81)	135.5(2)
O(2)-O(1)-C(1)-C(51)	-105.8(3)
C(3)-O(3)-C(2)-O(4)	-94.3(2)
C(3)-O(3)-C(2)-C(1)	28.9(3)
C(10)-O(4)-C(2)-O(3)	177.0(2)
C(10)-O(4)-C(2)-C(1)	52.8(3)
O(1)-C(1)-C(2)-O(3)	-55.5(3)
C(81)-C(1)-C(2)-O(3)	-170.0(2)
C(51)-C(1)-C(2)-O(3)	62.4(3)
O(1)-C(1)-C(2)-O(4)	63.3(3)

C(81)-C(1)-C(2)-O(4)	-51.2(3)
C(51)-C(1)-C(2)-O(4)	-178.8(2)
O(1)-O(2)-C(3)-O(3)	-72.0(3)
O(1)-O(2)-C(3)-C(11)	173.8(2)
O(1)-O(2)-C(3)-C(4)	48.9(3)
C(2)-O(3)-C(3)-O(2)	32.5(3)
C(2)-O(3)-C(3)-C(11)	144.9(3)
C(2)-O(3)-C(3)-C(4)	-90.4(3)
O(2)-C(3)-C(4)-C(5)	-92.3(4)
O(3)-C(3)-C(4)-C(5)	27.9(4)
C(11)-C(3)-C(4)-C(5)	148.4(3)
C(3)-C(4)-C(5)-C(51)	55.8(4)
C(12)-C(6)-C(7)-C(8)	-176.2(3)
C(51)-C(6)-C(7)-C(8)	-52.5(4)
C(6)-C(7)-C(8)-C(81)	58.2(4)
C(14)-N(1)-C(10)-O(4)	54.6(3)
C(16)-N(1)-C(10)-O(4)	-71.7(3)
C(14)-N(1)-C(10)-C(9)	-67.4(3)
C(16)-N(1)-C(10)-C(9)	166.2(3)
C(2)-O(4)-C(10)-N(1)	-175.4(2)
C(2)-O(4)-C(10)-C(9)	-51.6(3)
C(13)-C(9)-C(10)-N(1)	-64.5(3)
C(81)-C(9)-C(10)-N(1)	170.9(3)
C(13)-C(9)-C(10)-O(4)	174.7(3)
C(81)-C(9)-C(10)-O(4)	50.0(3)
C(10)-N(1)-C(14)-C(15)	173.0(2)
C(16)-N(1)-C(14)-C(15)	-58.0(3)
C(21)-N(2)-C(15)-C(14)	172.43(19)
C(17)-N(2)-C(15)-C(14)	-60.1(3)
N(1)-C(14)-C(15)-N(2)	60.0(3)
C(10)-N(1)-C(16)-C(17)	-173.8(2)
C(14)-N(1)-C(16)-C(17)	57.9(3)
C(21)-N(2)-C(17)-C(16)	-171.91(18)
C(15)-N(2)-C(17)-C(16)	59.5(3)
N(1)-C(16)-C(17)-N(2)	-59.0(3)
C(15)-N(2)-C(21)-O(20)	52.3(2)
C(17)-N(2)-C(21)-O(20)	-73.2(2)
C(15)-N(2)-C(21)-C(22)	-68.4(2)

C(17)-N(2)-C(21)-C(22)	166.03(19)
C(25)-O(20)-C(21)-N(2)	168.94(16)
C(25)-O(20)-C(21)-C(22)	-67.8(2)
N(2)-C(21)-C(22)-O(22)	-61.7(2)
O(20)-C(21)-C(22)-O(22)	176.49(17)
N(2)-C(21)-C(22)-C(23)	-179.19(18)
O(20)-C(21)-C(22)-C(23)	59.0(2)
O(22)-C(22)-C(23)-O(23)	73.0(2)
C(21)-C(22)-C(23)-O(23)	-166.67(18)
O(22)-C(22)-C(23)-C(24)	-167.76(17)
C(21)-C(22)-C(23)-C(24)	-47.4(2)
O(23)-C(23)-C(24)-O(24)	-73.8(2)
C(22)-C(23)-C(24)-O(24)	163.64(16)
O(23)-C(23)-C(24)-C(25)	164.41(17)
C(22)-C(23)-C(24)-C(25)	41.8(2)
C(21)-O(20)-C(25)-C(26)	-176.89(17)
C(21)-O(20)-C(25)-C(24)	61.6(2)
O(24)-C(24)-C(25)-O(20)	-167.27(17)
C(23)-C(24)-C(25)-O(20)	-47.6(2)
O(24)-C(24)-C(25)-C(26)	73.9(2)
C(23)-C(24)-C(25)-C(26)	-166.42(19)
O(20)-C(25)-C(26)-O(26)	-64.1(2)
C(24)-C(25)-C(26)-O(26)	57.2(2)
C(4)-C(5)-C(51)-C(6)	-167.4(3)
C(4)-C(5)-C(51)-C(1)	-38.8(4)
C(12)-C(6)-C(51)-C(5)	-62.2(4)
C(7)-C(6)-C(51)-C(5)	174.4(3)
C(12)-C(6)-C(51)-C(1)	169.9(3)
C(7)-C(6)-C(51)-C(1)	46.5(4)
O(1)-C(1)-C(51)-C(5)	71.9(3)
C(2)-C(1)-C(51)-C(5)	-48.5(4)
C(81)-C(1)-C(51)-C(5)	-175.0(3)
O(1)-C(1)-C(51)-C(6)	-160.4(3)
C(2)-C(1)-C(51)-C(6)	79.2(4)
C(81)-C(1)-C(51)-C(6)	-47.3(4)
C(7)-C(8)-C(81)-C(9)	176.0(3)
C(7)-C(8)-C(81)-C(1)	-57.8(3)
C(10)-C(9)-C(81)-C(8)	73.2(4)

C(13)-C(9)-C(81)-C(8)	-49.0(4)
C(10)-C(9)-C(81)-C(1)	-53.1(4)
C(13)-C(9)-C(81)-C(1)	-175.3(3)
O(1)-C(1)-C(81)-C(8)	166.1(2)
C(2)-C(1)-C(81)-C(8)	-76.0(3)
C(51)-C(1)-C(81)-C(8)	52.3(3)
O(1)-C(1)-C(81)-C(9)	-65.7(3)
C(2)-C(1)-C(81)-C(9)	52.2(3)
C(51)-C(1)-C(81)-C(9)	-179.5(3)

Symmetry transformations used to generate equivalent atoms:

Table S13. Hydrogen bonds for **18** (yuet5) [Å and °].

D-H...A	d(D-H)	d(H...A)	d(D...A)	<(DHA)
O(22)-H(22D)...O(1W)	0.85	1.93	2.741(2)	157.9
O(23)-H(23D)...O(22)#1	0.85	2.15	2.915(2)	149.5
O(24)-H(24D)...O(23)#2	0.85	2.17	2.978(2)	159.0
O(1W)-H(1W)...O(26)#3	0.85	1.96	2.798(3)	169.1
O(1W)-H(2W)...O(24)#1	0.85	2.11	2.942(3)	165.4

Symmetry transformations used to generate equivalent atoms:

#1 -x,y-1/2,-z #2 -x-1,y+1/2,-z #3 x+1,y,z
